# RBM47 inhibits hepatocellular carcinoma progression by targeting UPF1 as a DNA/RNA regulator

**DOI:** 10.1038/s41420-022-01112-3

**Published:** 2022-07-14

**Authors:** Tao Guo, Ke You, Xi Chen, Yuqi Sun, Ying Wu, Ping Wu, Yingying Jiang

**Affiliations:** 1 Department of Pathophysiology, School of Basic Medical Sciences, Weifang Medical University, Weifang, 261053 China; 2 Department of Hepatobiliary Surgery, The Second Affiliated Hospital of Chongqing Medical University, Chongqing, 400010 China; 3 School of Stomatology, Weifang Medical University, Weifang, 261053 China; 4 School of Clinical Medicine, Weifang Medical University, Weifang, 261031 China; 5 Liuzhou Key Laboratory of Infectious Disease Immunity Research, Guangxi Health Commission Key Laboratory of Clinical Biotechnology, Liuzhou People’s Hospital affiliated to Guangxi Medical University, Liuzhou, 545006 China; 6 Department of Pediatric Surgery, Guangzhou Institute of Pediatrics, Guangzhou Women and Children’s Medical Center, Guangzhou Medical University, Guangzhou, 510000 China; 7 Department of Dentistry, Affiliated Hospital of Weifang Medical University, Weifang, 261035 China

**Keywords:** Tumour-suppressor proteins, Tumour-suppressor proteins

## Abstract

The mechanisms by which the tumor behaviors of hepatocellular carcinoma (HCC) support growth and metastasis remain largely unknown, and it has become increasingly apparent that molecular dysregulation is of considerable importance for cellular signaling pathways. Recently, RNA-binding motif protein 47 (RBM47) has been suggested to function as a tumor regulator by acting as an RNA binding protein (RBP), but its role in HCC remains ambiguous. Here, in HCC, we identified that RBM47 had an inhibitory influence on tumor behaviors in vitro and accordingly suppressed the growth and metastasis of xenograft tumors in vivo. Additionally, RBM47 was verified to positively regulate Upframeshift 1 (UPF1), which is a crucial protein involved in the nonsense-mediated RNA decay (NMD) process and was previously determined to be an HCC suppressor. Mechanistically, the stability of UPF1 mRNA was demonstrated to be enhanced with its 3’UTR bound by RBM47, which acted as an RNA binding protein. Meanwhile, RBM47 was also proven to promote the transcription of UPF1 as a transcription factor. Taken together, we concluded that RBM47 functioned as a tumor suppressor by upregulating UPF1, acting as a DNA/RNA binding protein at the transcriptional and posttranscriptional levels.

## Introduction

Hepatocellular carcinoma (HCC) is one of the most prevalent malignancies and a principal cause of cancer-related mortality throughout the world [1]. Treatment approaches for HCC are limited, in part owing to the insufficient knowledge of the fundamental molecular mechanisms underlying HCC [2, 3]. The progression of HCC is a multistep and complicated process involving both environmental and genetic factors [4]. Normally, the regenerative lesions in cirrhosis create an appropriate microenvironment for the transformation of normal hepatocytes to dysplastic hepatocytes and neoplastic lesions, eventually leading to HCC by further epigenetic and genetic alterations [5, 6]. In the meantime, molecular disorders in HCC are commonly detected at the nucleic acid and protein levels. And uncontrolled RNA decay or activation was discovered in HCC triggering or progressing through RNA binding protein (RBP) participation [7]. Therefore, RBP-mediated RNA modifications are crucial for cancer development, and abnormal RBP expression in cancers can regulate the expressions and functions of oncogenes and tumor suppressor genes [8, 9].

The crucial biological roles of the RNA-binding motif protein (RBMP) family have recently been revealed. As RBPs, RBMPs play important roles in the regulation of RNA editing, RNA modification, alternative splicing (AS), and mRNA stability [10, 11]. Moreover, accumulating evidence suggests that abnormal expression levels of RBMPs are liked to cancer development and progression [12, 13]. We have recently learned that the dysregulation of the RBMP RNA-binding motif protein 47 (RBM47), which contributes to various mRNA-related pathways, is related to cancer [14]. Interestingly, RBM47 has opposing roles in different cancers, although it mainly functions as an RBP and may even exert regulatory effects on DNA [14–16]. However, its functions in HCC progression as well as its underlying mechanisms of action remain largely uncertain.

On the other hand, as a master regulator of nonsense-mediated mRNA decay (NMD) [17], Up-frameshift protein 1 (UPF1) is a tumor regulator and a potential biomarker [18]. In HCC, UPF1 acts as an HCC suppressor [19, 20]. In this study, we characterized the inhibitory role of RBM47 in HCC via UPF1. In particular, we evaluated the function and mechanism of action of RBM47 in the regulation of HCC in vitro and in vivo. Our findings highlight the multifaceted roles of RBM47 in HCC.

## Results

### RBM47 inhibits HCC progression in vitro

**Fig. 1:**
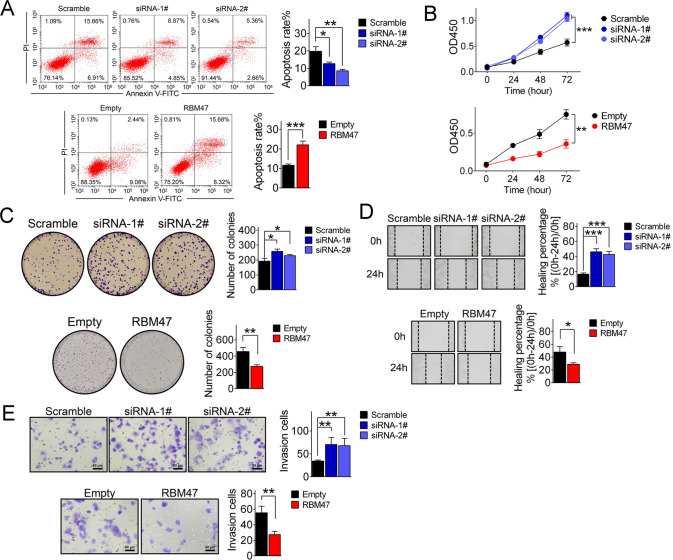
RBM47 inhibits oncogenic behaviors in vitro. **A** Flow cytometric analysis determined that RBM47 could significantly promote apoptosis in Huh7 (upper panel) and HCCLM3 (lower panel) cells. Error bars are SD (*n* = 3). **B** Proliferation of Huh7 (upper panel) and HCCLM3 (lower panel) cells was tested by CCK-8 assay after RBM47 knockdown and overexpression, respectively. Error bars are SD (*n* = 3). **C** Colony formation was enhanced when RBM47 expression was decreased (upper panel) and suppressed when RBM47 expression was enhanced (lower panel) in Huh7 and HCCLM3 cells. Error bars are SD (*n* = 3). **D** Representative images of wound healing assays performed after RBM47 silencing in Huh7 cells (upper panel) and enforced overexpression in HCCLM3 cells (lower panel). Error bars are SD (*n* = 3). **E** Transwell assays performed using Huh7 cells after RBM47 knockdown and HCCLM3 cells after RBM47 overexpression (bar = 60 μm). Error bars are SD (*n* = 3). *P < 0.05, **P < 0.01, ***P < 0.001.

To determine the regulatory roles of RBM47 in HCC progression, in vitro analyses of oncogenic behavior were performed. Firstly, we examined the expression of RBM47 in different hepatoma cell lines by qRT-PCR and noticed that RBM47 presented higher expression in Huh7 cells and lower expression in HCCLM3 cells (Supplementary Fig. 1A). We then knocked down RBM47 in Huh7 cells and enforced enhancement in HCCLM3 cells (Supplementary Fig. 1B, C) for analyses of the rate of apoptosis by flow cytometry. RBM47 expression resulted in an increased rate of apoptosis in hepatoma cells (Fig. 1A). Meanwhile, we tested the proliferation capacity and cell growth potential by CCK-8 and colony formation assays after RBM47 knockdown and overexpression in Huh7 and HCCLM3 cells. It was discovered that RBM47 could significantly suppress hepatoma cell proliferation and growth (Fig. 1B, C). Additionally, the findings of wound healing and Transwell assessments illuminated that migration and invasion were enhanced when RBM47 was silenced and were repressed after RBM47 overexpression (Fig. 1D, E). The achieved outcomes divulged that upregulation of RBM47 could remarkably suppress HCC progression in vitro.

### RBM47 suppresses tumor growth and metastasis in vivo

**Fig. 2:**
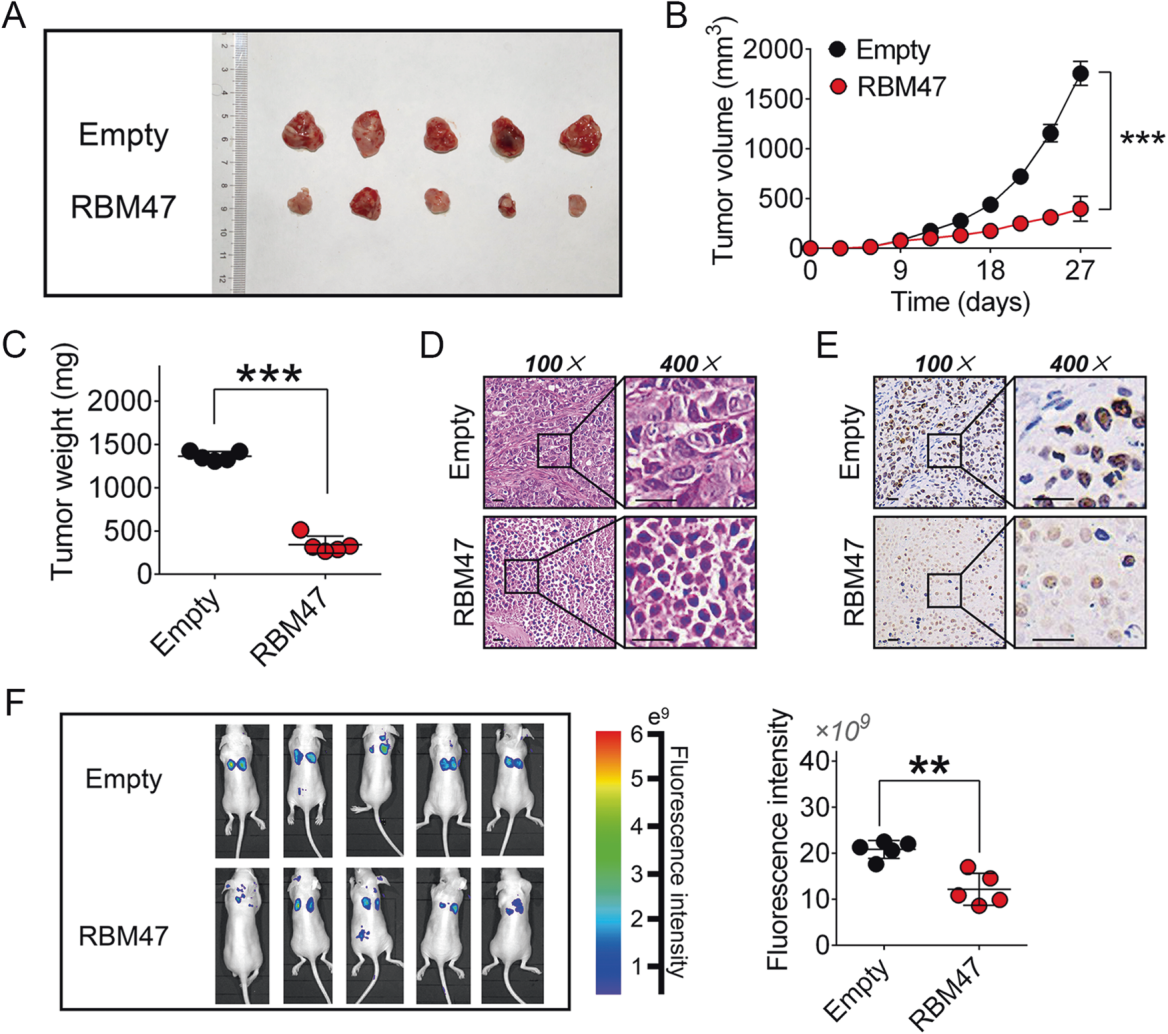
RBM47 inhibits tumor growth and metastasis in vivo. **A** Representative images of removed subcutaneously implanted tumors. **B** The effects of RBM47 on tumor growth are summarized by a tumor growth curve. Error bars are SD (*n* = 5). **C** Comparison of tumor weight between the RBM47 overexpression group and the control group. Error bars are SD (*n* = 5). Microscopic images of HE (**D**) and immunohistochemical staining patterns for Ki-67 (**E**) in tumor xenografts from the nude mice in the RBM47 overexpression group and the control group (scale bars = 50 μm). **F** Observation of lung metastasis by in vivo imaging after tail vein injection with cells stably transfected with RBM47 or empty vector with fluorescence labeling (left panel) and quantitative analysis of fluorescence intensity (right panel). Error bars are SD (*n* = 5). **P < 0.01, ***P < 0.001.

Tumor growth and metastatic models were generated through implementing HCCLM3 cells stably transfected with RBM47 containing fluorescent labeling (Supplementary Fig. 2). After subcutaneous injection and sacrifice, the subcutaneous tumor was removed (Fig. 2A). The curve of tumor growth and weight of the implanted tumor demonstrated that RBM47 could successfully limit the tumor size (Fig. 2B, C). HE and IHC analyses indicated that Ki-67 expression was reduced when RBM47 levels increased (Fig. 2D, E). We then observed tumor metastasis in the lungs by in vivo fluorescent imaging and discovered that RBM47 overexpression could significantly reduce hepatoma cell metastasis in vivo (Fig. 2F).

### UPF1 is the target gene of RBM47

**Fig. 3:**
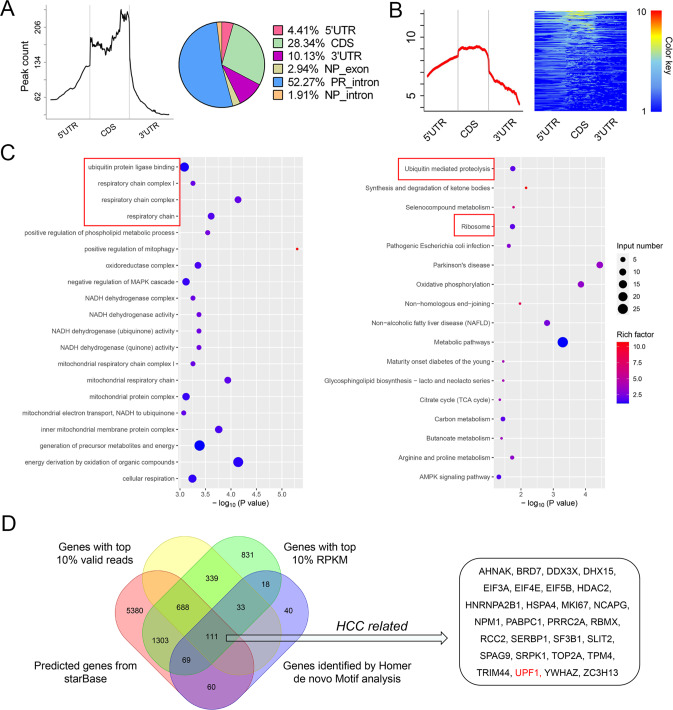
Identification of the target gene of RBM47. **A** Peak distribution across the 5’UTR, CDS, and 3’UTR (left panel) as well as other functional segments (right panel). **B** Distribution of reads in peak regions. **C** GO (left) and KEGG (right) pathway analyses of genes predicted to bind to RBM47. The red box indicates pathways predicted to involve UPF1. **D** Venn diagram of target genes of RBM47 based on online prediction using starBase and RIP-sequencing.

**Fig. 4:**
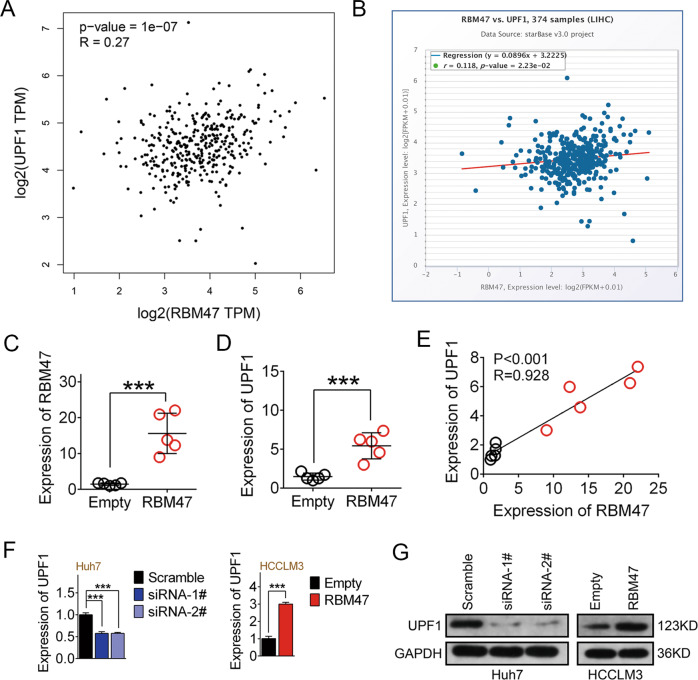
RBM47 upregulates UPF1 in HCC. **A** Bivariate correlation analysis of the relationship between RBM47 and UPF1 in HCC tissues in the GEPIA database. **B** A positive correlation between RBM47 and UPF1 was determined in 374 HCC tissues in starBase database. **C**–**E** RBM47 expression, UPF1 expression, and their correlation of subcutaneously implanted tumors between the RBM47 overexpression group and the control group. Red dots represent the RBM47 overexpression group, and black dots represent the control group. Error bars are SD (*n* = 5). **F**, **G** qRT-PCR and western blot analysis of the expression of UPF1 after RBM47 knockdown in Huh7 cells and overexpression in HCCLM3 cells. ***P < 0.001.

RBM47 plays an inhibitory role in HCC progression both in vivo and in vitro, however, the fundamental molecular mechanism underlying these effects remains uncertain. To identify the target gene of RBM47, we predicted target genes using starBase database (https://starbase.sysu.edu.cn/) [21] yielding 7611 candidates. We then conducted RIP-seq to identify the RNAs binding to RBM47. The RBM47-3×Flag (RBM47^Flag^) fusion protein was constructed in HCCLM3 cells for RIP and subsequent RNA sequencing revealed 11724 valid sequences among 56304 candidates. By comprehensively analyzing the results of RIP-seq, 681 sequences were addressed by Peak Calling analysis, and 30 (4.41%), 193 (28.34%) and 69 (10.13%) of peaks were distributed in 5’UTR, CDS and 3’UTR, respectively (Fig. 3A). Meanwhile, it was discovered that the distributions of peaks and associated reads were basically consistent (Fig. 3B). GO and KEGG analyses indicated corresponding pathways in downstream pathway regulation (Fig. 3C). Furthermore, the sequences with top 10% of valid reads, top 10% of RPKM and RNA levels in a novo motif analysis using Homer were selected as candidate target genes. After taking the intersection of genes identified by each approach including the prediction in starBase, we obtained 111 RNAs predicted to interact with RBM47, including 28 related to HCC (Fig. 3D). Taking into consideration of the enrichment of GO and KEGG, the Upframeshift 1 (UPF1), which is a tumor suppressor in HCC [19, 20, 22], was predicted to be a direct target of RBM47 (Fig. 3C, D). To verify that UPF1 is a functional target of RBM47, we first detected the association between RBM47 and UPF1 in HCC tissues using the GEPIA databank (http://gepia.cancer-pku.cn/index.html) and starBase [21, 23]. The results indicated that the expression of RBM47 and UPF1 was positively correlated in human HCC tissues (Fig. 4A, B). Then, we tested RBM47 and UPF1 expression in subcutaneously implanted tumors from the abovementioned mouse model. Both RBM47 and UPF1 were upregulated in subcutaneous tumors stably transfected with RBM47 (Fig. 4C, D), and their expression levels were significantly positively correlated (Fig. 4E). We next knocked down RBM47 in Huh7 cells and overexpressed RBM47 in HCCLM3 cells and discovered that UPF1 was positively regulated by RBM47, as determined by qRT-PCR and western blotting (Fig. 4F, G).

### RBM47 inhibits HCC progression by upregulating UPF1

**Fig. 5:**
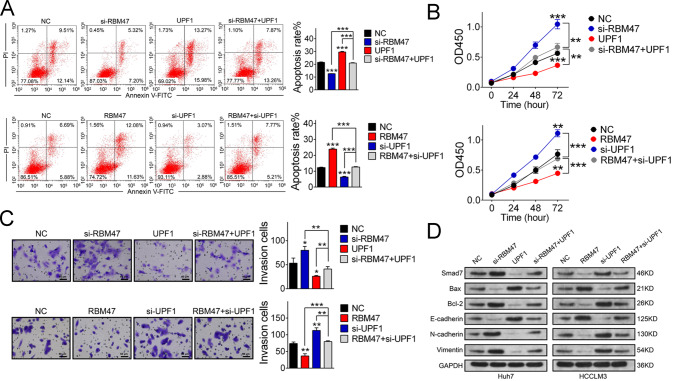
RBM47 inhibited HCC through regulating UPF1. **A**–**C** Flow cytometric analysis, CCK-8 assay, and Transwell assay (bar = 60 μm) after RBM47 knock down and UPF1 upregulation in Huh7 cells, or enhancing RBM47 and silencing UPF1 in HCCLM3 cells. Error bars are SD (*n* = 3). **D** Rescue experiments of effects of RBM47 on target genes regulated by UPF1 by western blotting. Error bars are SD (*n* = 3). *P < 0.05, **P < 0.01, ***P < 0.001.

We know that UPF1 behaves as a tumor suppressor in multiple ways through targeting different tumor-associated proteins [19, 22, 24]. To elucidate whether RBM47 inhibited HCC progression via UPF1, we knocked down RBM47 and overexpressed the UPF1 (Supplementary Fig. 3) in Huh7 cells. We also evaluate apoptosis in HCCLM3 cells with increased RBM47 expression and silenced UPF1 (Supplementary Fig. 3) by flow cytometry. In rescue experiments, the rate of apoptosis decreased in response to the silencing of either RBM47 or UPF1 (Fig. 5A). We next investigated the effects of RBM47 and UPF1 on cell proliferation by CCK-8 assay. The results exhibited that both RBM47 and UPF1 were crucial for cell proliferation (Fig. 5B). Additionally, the results of rescue experiments indicated that RBM47 repressed the cell invasion via UPF1, as determined by a Transwell assay (Fig. 5C). Therefore, we may speculate that RBM47 could inhibit HCC tumor cell processes by increasing UPF1. To further verify this hypothesis, we performed rescue experiments by the expression of tumor-associated proteins regulated by UPF1 in HCC. UPF1 was enhanced in Huh7 cells and silenced in HCCLM3 cells with RBM47 overexpression or knockdown, and the results of rescue experiments revealed that RBM47 could regulate tumor-associated proteins, which were also regulated by UPF1, as determined by western blotting (Fig. 5D). Thus, we deduced that RBM47 could inhibit HCC progression by upregulating UPF1.

### RBM47 upregulated UPF1 by binding to its 3’UTR

**Fig. 6:**
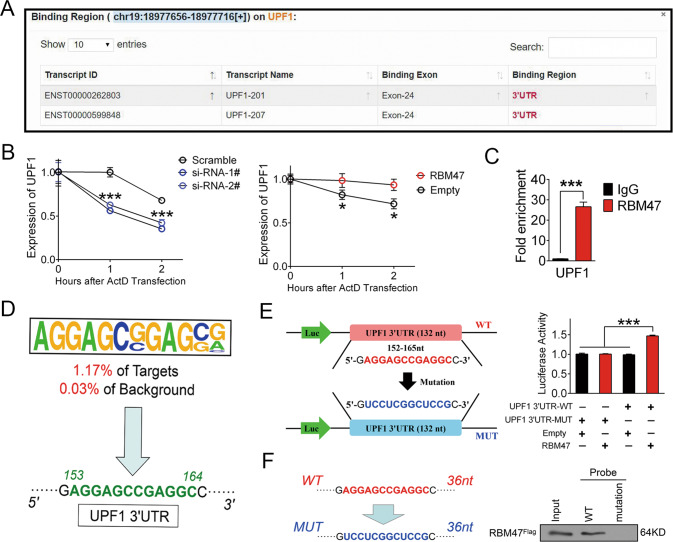
RBM47 upregulated UPF1 by binding its 3’UTR. **A** Online screenshot of binding site of RBM47 in UPF1 mRNA at starBase 3.0. **B** RNA decay assay was performed by ActD treatment in Huh7 cells after RBM47 knockdown (left panel) and overexpression in HCCLM3 cells (right panel). Error bars are SD (*n* = 3). **C** The direct binding of RBM47 and UPF1 mRNA was detected by RIP-PCR in HCCLM3 cells. **D** The preferred sequence of the UPF1 3’UTR was identified by motif analysis according to known gene matching. **E** The effect of RBM47 on the UPF1 3’UTR was tested by a luciferase reporter assay with a 3’UTR mutation vector. Error bars are SD (*n* = 3). **F** The direct binding locus was determined by the RNA pulldown assay with a mutant 36 nt probe with predicted binding sites. *P < 0.05, ***P < 0.001.

To further understand the mechanisms by which RBM47 regulates UPF1, we predicted the binding site of RBM47 on UPF1 using starBase. A bioinformatics analysis suggested that RBM47 interacts with UPF1 mRNA and directly binds to its 3’UTR (Fig. 6A). We understand that RBPs may bind to the 3’UTR of target genes to enhance stability [25]. Therefore, we speculated that RBM47 may upregulate UPF1 by enhancing mRNA stability by binding its 3’UTR. To verify this speculation, we conducted an RNA decay assay in hepatoma cells treated with ActD. The results indicated that UPF1 mRNA degradation was accelerated with RBM47 silencing and stabilized after RBM47 overexpression (Fig. 6B). We next perform RIP-PCR to determine the direct binding of RBM47 to UPF1 mRNA. The RIP-PCR results implied that UPF1 mRNA could be enriched by RBM47 (Fig. 6C). Additionally, Homer de novo Motif analysis was performed to evaluate the region targeted by RBM47 in the 3’UTR of UPF1. With observing the addressed motifs from RIP-seq (Supplementary Table 3), we noticed the a sequence AGGAGCCGAGGC, which possessed 1.17% predicted targets and 0.03% backgrounds, was matched in the 3’UTR of UPF1 (Fig. 6D). We then compared the specific sequence in the UPF1 3’UTR (basically composed of the 24th exon) and discovered its existence and initiation-end site (*153–164*th locus from the start site of the 3’UTR) (Fig. 6D). Thus, we generated UPF1 3’UTR vectors including wild-type and mutant UPF1 to perform luciferase reporter assays in HCCLM3 cells and found that upregulation of RBM47 enhanced the activity of the UPF1 3’UTR (Fig. 6E). Finally, we also generated an RNA probe with mutation of the putative binding site to perform an RNA pulldown assay in HCCLM3 cells, and the results proved that the wild-type UPF1 3’UTR probe could pull down the RBM47^Flag^ fusion protein, while the mutation could not (Fig. 6F). Therefore, we concluded that RBM47 upregulated UPF1 by binding its 3’UTR to stabilize its mRNA.

### RBM47 upregulates the level of UPF1 precursor mRNA (pre-mRNA) in the nucleus

**Fig. 7:**
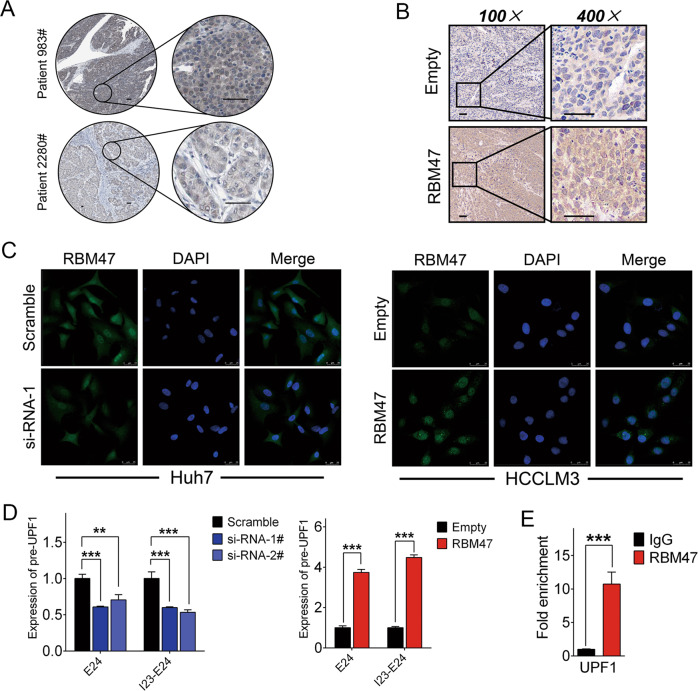
RBM47 enhanced level of the RBM47 pre-mRNA. The subcellular localization of RBM47 was observed in HCC tissues (**A**) at HPA databank (referred scale bars = 48 μm), subcutaneously implanted tumors of nude mice (**B**) by IHC (scale bars = 50 μm), and in hepatoma cells (**C**) by IF after RBM47 silencing or enforced enhancement (scale bars = 25 μm). **D** The intranuclear UPF1 pre-mRNA was tested by qRT-PCR in Huh7 cells with RBM47 knockdown and in HCCLM3 cells with RBM47 overexpression. Error bars are SD (*n* = 3). **E** ChIP experiments were performed in HCCLM3 cells to detect the direct binding between RBM47 and the UPF1 promoter, and promoter enrichment was tested by qRT-PCR. Error bars are SD (*n* = 3). **P < 0.01, ***P < 0.001.

We have demonstrated the posttranscriptional effects of RBM47 on UPF1 mRNA based on bioinformatics and in vitro experiments. Notably, RBM47 also has DNA binding potential as a transcriptional regulator [15, 26], which may synergistically upregulate UPF1 mRNA at different levels. To address our deduction, we online observed the subcellular localization of RBM47 in HCC tissues at Human Protein Atlas (HPA) databank (https://www.proteinatlas.org/) and noticed that RBM47 was distributed in nucleus and cytoplasm (Fig. 7A). We then performed IHC and IF to further observe the subcellular distribution of the RBM47 protein. The results indicated that the RBM47 protein was concurrently located in both the nucleus and cytoplasm in subcutaneously implanted tumor tissue and hepatoma cells (Fig. 7B, C). Next, we tested the effects of RBM47 on the pre-3’UTR of UPF1, which is also its 24th exon in the nucleus. We generated primers that span the junction between the 23rd intron and 24th exon to test the pre-mRNA of UPF1 in the nucleus in parallel by qRT-PCR. The achievements illustrated that RBM47 could positively regulate the expression of the 24th exon and segment containing the 23rd intron and 24th exon in the nucleus, which means that RBM47 could enhance the level of UPF1 pre-mRNA and may perform an essential role at the transcriptional level (Fig. 7D). Next, we aimed to confirm the direct binding between RBM47 and the UPF1 promoter, and the ChIP-PCR results demonstrated that RBM47 enriched the segments of the UPF1 promoter (Fig. 7E). Based on these results, we concluded that RBM47 could upregulate UPF1 pre-mRNA at the transcriptional level.

### RBM47 promotes UPF1 transcription by acting as a DNA binding protein

**Fig. 8:**
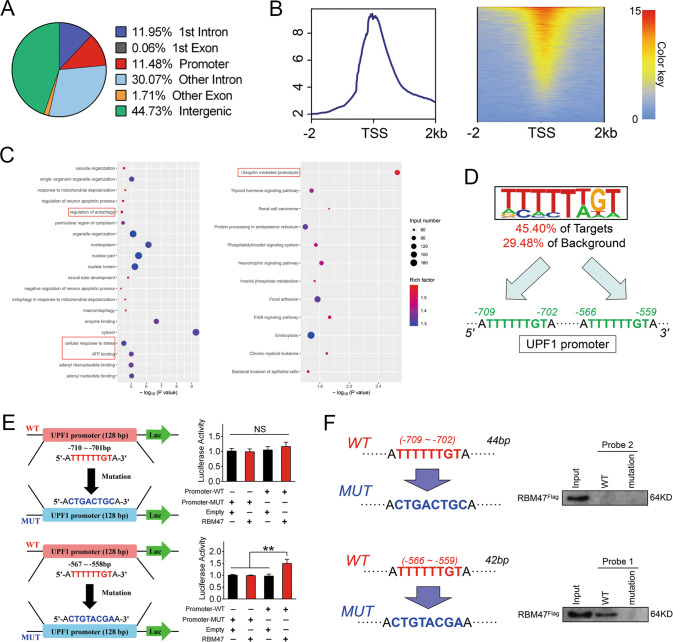
RBM47 promoted UPF1 transcription by binding its promoter. **A** The pie chart presents the peak distribution on the chromosome in detail. **B** Distribution of peak-associated reads on both sides of the transcription initiation site (TSS) within 2000 bp upstream and downstream. **C** GO (left) and KEGG (right) pathway analyses of binding genes. The red box indicates pathways that may involve UPF1. **D** Common binding loci were addressed on the UPF1 promoter after motif analysis based on novel gene matching. **E** Luciferase activities were determined by dual luciferase analysis to evaluate the promoting effects of RBM47 on UPF1. Error bars are SD (*n* = 3). **F** Western blot analysis of the specific association of the UPF1 promoter with RBM47^Flag^ in DNA pulldown assays with 2 mutant probes. NS, not significant, **P < 0.01.

**Fig. 9:**
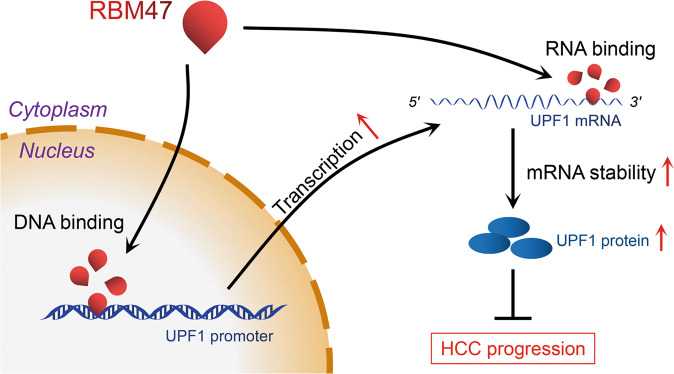
Schematic of the proposed mechanism by which RBM47 inhibits HCC progression. As an RNA binding protein, RBM47 elevated UPF1 mRNA by binding to the UPF1 3’UTR to enhance its stability. Meanwhile, RBM47 directly bound to the promoter of UPF1 as a DNA binding protein to promote UPF1 transcription. Thus, RBM47 suppressed HCC progression by enhancing UPF1 as a DNA/RNA regulator. HCC Hepatocellular carcinoma, RBM47 RNA-binding motif protein 47, RBP RNA binding protein, UPF1 Up-frameshift 1, NMD nonsense-mediated RNA decay, TF transcription factor, IHC Immunohistochemistry, IF Immunofluorescence, RIP RNA immunoprecipitation, RPKM Reads Per Kilo bases per Million reads, ChIP Chromatin immunoprecipitation, pre-mRNA precursor mRNA, TSS transcription initiation site, RRM RNA recognition motif, AS alternative splicing.

Above results demonstrated that RBM47 could regulate UPF1 at the transcriptional level. However, the specific binding site of RBM47 on UPF1 promoter was not identified. Thus, DNA sequencing was conducted to analyze the specific loci binding by RBM47 after the ChIP in HCCLM3 cells. By analyzing the data of ChIP-seq, we discovered that the promoter-associated sequences were enriched in 11.48% of peaks, and functional reads were basically evenly distributed in the upstream and downstream sections of transcription initiation site (TSS) (Fig. 8A, B). We then discovered that the pathways involving UPF1 were also enriched by GO and KEGG analysis (Fig. 8C). To determine the DNA binding sequences of RBM47, motif analysis was performed. The results of Homer de novo Motif analysis revealed preferred motifs (Supplementary Table 4) and 2 preferred loci with same sequence TTTTTTGT were found in the UPF1 promoter (Fig. 8C). To verify the predicted binding locus in the UPF1 promoter, we conducted a luciferase reporter assay by transfecting the UPF1 promoter with putative mutant binding sites in HCCLM3 cells. Interestingly, we discovered that RBM47 promoted the activity of the UPF1 promoter with binding sites of −559 to −566 (Fig. 8E). Additionally, we generated wild-type DNA probes and 2 mutation probes to perform DNA pulldown analysis in HCCLM3 cells. The results determined that the loci of −559 to −566 were the direct binding sites of RBM47 on the UPF1 promoter, which was consistent with the luciferase reporter assay results (Fig. 8F). Therefore, we demonstrated that RBM47 inhibited HCC progression by upregulating UPF1 by promoting its transcription and mRNA stability as a DNA/RNA regulator (Fig. 9).

## Discussion

Current research has determined the role of RBM47 in HCC progression, which presents inhibitory effects on HCC both in vitro and in vivo in multiple ways. Moreover, our results verified that RBM47 inhibited HCC progression by positively regulating UPF1. Mechanistically, RBM47 could bind to the UPF1 3’UTR and its promoter to increase the expression of UPF1. These results clearly indicated that RBM47 promoted UPF1 transcription and enhanced the stability of UPF1 mRNA by DNA/RNA binding to inhibit HCC progression.

Human RBM47 (also known as NET18) is mapped to chromosomal location 4p14 on the reverse strand, and its full-length transcript contains 23 introns and 24 exons. RBM47 was discovered as a differentially expressed gene in the foregut endoderm of the E8.5 mouse embryo [27] and then revealed to be imperative for vertebrate embryonic growth and survival [28, 29]. In cancers, the aberrant expression of RBM47 influences the transcriptional or posttranscriptional regulation of multiple tumor-suppressor and proto-oncogenic targets across diverse cancer types. For instance, the dysregulation of RBM47 in various tumors is associated with a poor prognosis [30–32]. Functionally, RBM47 was first identified as a tumor suppressor in breast cancer. RBM47 is significantly downregulated in claudin-low and basal breast cancers with heterozygous or homozygous loss of functions and during lung and brain metastasis [16]. RBM47 has also been recognized as a posttranscriptional regulator of epithelial cell-specific alternative splicing events and as a modulator of mRNA stability for key Wnt/β-catenin signaling antagonists, through which it exerts tumor-suppressive effects [33]. In our study, we also identified the performance of RBM47 as a tumor suppressor in HCC, including inhibiting tumor growth and metastasis. Moreover, the depletion of RBM47 led to the downregulation of epithelial-associated genes including OCLN, CDH1, TJP1, and CLDN1 with a concomitant enhancement in cell migration, mesenchymal markers, invasion and metastasis in colorectal cancer [30], which was consistent with our report. Additionally, an antagonistic correlation of RBM47 with TGF-β signaling has already been explained, whereby RBM47 hinders TGF-β-induced nuclear factor erythroid 2-related Factor 2 (NRF2) activity through stabilizing the mRNAs of its negative regulators, viz. CUL3 and KEAP1 and improving their protein levels [34]. Therefore, RBM47 is commonly regarded as a functional tumor suppressor in multiple cancer types. As an RBP, RBM47 contains three RNA recognition motifs (RRMs) and an alanine-rich region at its C-terminus [14, 35]. In addition, as an RBP, unknown original motifs have not been comprehensively addressed, resulting in different molecular roles of RBM47 in regulating RNAs. As mentioned above, RBM47 is a recently discovered posttranscriptional regulator of epithelial cell-specific alternative splicing events [33]. At the same time, RBM47 also partially applies its tumor-suppressive functions through modulating mRNA stability by 3’UTR binding [16, 36, 37]. By RIP sequencing, we also determined that RBM47 modulated UPF1 by stabilizing its mRNA and addressed the novel motif in the UPF1 3’UTR with direct binding. Consistent with previous investigations, RBM47 had a tumor-suppressive function in HCC via 3’UTR binding. Meanwhile, we also addressed the DNA binding function of RBM47 acting as a transcription factor on UPF1, and we elucidated the DNA binding motif by DNA sequencing. At transcriptional regulation, RBM47 may reveal an ambiguous role in cancer. Our results demonstrated the tumor-suppressive effects of RBM47 in HCC, and a previous investigation also determined the positive transcriptional control of RBM47 on p53, which is a recognized tumor suppressor [26]. However, in nasopharyngeal carcinoma, RBM47 presented oncogenic effects on tumor behaviors by acting as a DNA/RNA binding protein [15]. Additionally, RBM47 may present malignant significance on survival [15, 32]. These seemingly contradictory conclusions may be related to tumor heterogeneity, pathological types or molecular mechanisms resulting in disease specificity.

We provide the first evidence for the role of RBM47 in HCC and made detailed elucidation of its molecular mechanisms by targeting UPF1. UPF1 is an RNA helicase taking part in almost all steps of the NMD procedure, from target discrimination to remodeling the complex of NMD-mRNA and improving nuclease accessibility to the mRNA. Thus, UPF1 is considered to mediate the degradation of the substrates of NMD via a phosphorylation/dephosphorylation cycle [38, 39]. In tumorigenesis, recent investigations have demonstrated the inhibitory role of UPF1 in tumor progression in multiple cancers [40–42]. In HCC, UPF1 was demonstrated to act as a tumor suppressor via multiple pathways. For example, UPF1 was determined to promote cell apoptosis and enhance sensitivity to sorafenib [19]. Previous research also revealed its effects on suppressing cell invasion and metastasis by targeting Smad7 [22]. Interestingly, RBM47 expression failed to show clinical significance in analyses of data from GEPIA and starBase, although it was positively correlated with UPF1 in HCC. However, there is evidence that UPF1 acts as a tumor suppressor in HCC [19, 20, 22], and RBM47 reveals clinical value in some other cancers [15, 16, 30, 34]. The current lack of clinical value of RBM47 expression may be explained by sample selection, sample size, pathologic staging, etc. Therefore, further studies of the clinical role of RBM47 in HCC are needed. In this investigation, we objectively proved that UPF1 was the direct target gene of RBM47 at the transcriptional and posttranscriptional levels and concluded that RBM47 could inhibit HCC progression by upregulating UPF1.

RBM47 is a multifunctional protein that not just presents an RNA binding effect. According to its cellular distributions and previous investigations, RBM47 may play multiple roles in the process of molecular events [15, 26]. In current study, we determined that RBM47 could exert tumor suppressive effects via UPF1 and we identified several DNA/RNA binding motifs by sequencing. However, consensus nucleic acid sequences should be further explored. For example, the results of RIP-seq indicated a relative preference of CDS binding by RBM47, but RBM47 up regulated UPF1 through 3’UTR binding. Although we proved that RBM47 could stabilize UPF1 mRNA through 3’UTR binding, it was undeniable that RBM47 may regulate other genes through CDS or 5’UTR binding. The consensus sequences distributed in different binding regions need to be further addressed and studied. And the preferences of motif bindings of RBM47 in different regions also remain unknowns. Therefore, although we determined that RBM47 could exert tumor suppressive effects via UPF1, it may have additional targets in HCC. For DNA binding, the motifs seemed to remain probabilistic binding. Therefore, the putative two DNA binding sites we predicted in the current study have not been positively verified, and this may be influenced by other transcriptional cofactors, sites of helix or distance from the TSS. Notably, only 11.48% of peaks called by RBM47 was distributed in the promoter region according to the results of ChIP-seq, while it was unclear whether the bindings of other regions, such as the intergenic region, would lead to different gene regulation consequences. In addition, other molecular events based on DNA/RNA binding were not further investigated in the current research, either. For instance, although we demonstrated that RBM47 could enhance the stability of UPF1 mRNA by binding to the 3’UTR, we did not investigate the loci for RNA binding motifs in introns of pre-mRNA, which may reveal the function of RBM47 in posttranscriptional alternative splicing. Meanwhile, we did not evaluate the effects of RBM47 on the UPF1 enhancer with DNA binding motif detection. Clinically, the expression levels of RBM47 in clinical tissues have not been extensively evaluated to date, limiting our understanding of the clinical value of RBM47 in HCC to some extent.

Taken together, our results demonstrate that RBM47 functions as an effective suppressor of tumors by promoting the upregulation of UPF1. In future studies aimed at obtaining detailed insight into these and other RBM47 functions, other target genes and relative molecular events should be further addressed. Furthermore, it will be essential to scrutinize the links between our outcomes and clinical significance in a large-scale clinical setting.

## Materials and methods

### Cell culture

Hepatoma cell lines were obtained from the Cell Bank of Type Culture Collection (Chinese Academy of Sciences, Shanghai, China). Hepatoma cells were cultivated in minimum essential medium (Gibco) supplemented with 10% fetal bovine serum (Gibco), in accordance with recommendations, in an incubator with 5% CO_2_ at 37 °C.

### Cell transfection

To knock down RBM47 and UPF1, small interfering RNAs (siRNAs) targeting RBM47 or UPF1 were produced and examined through GeneCopoeia (GeneCopoeia, Inc.). The cells were transfected with 24 nmol/L siRNA employing Lipofectamine RNAiMAX reagent (Invitrogen) according to the protocol of the manufacturer. Meanwhile, pTT5 plasmid vectors loaded with UPF1 and RBM47 were also generated for overexpression. 3 × Flag tag amino acids (DYKDDDDK) were also fused at the end of RBM47 to construct the RBM47^Flag^ fusion protein. All plasmid vectors were transfected with Lipofectamine 2000 (Thermo Fisher) following the protocol of the manufacturer.

### RNA and DNA extraction

The extraction of the total cellular RNA was executed by employing TRIzol reagent (Invitrogen) in accordance with the protocol of the manufacturer. In addition, the isolation and purification of nuclear RNA and cytoplasmic were conducted by employing the Cytoplasmic and Nuclear RNA Purification Kit (Norgen, CA) in accordance with the instructions of the manufacturer. Extracted total RNA was quantified by implementing a NanodropTM spectrophotometer (Thermo Fisher) at 280 and 260 nm. RNA was employed for reverse transcription if A260/A280 ≥ 2.0. Total RNA was employed for synthesizing first-strand cDNA by implementing random primers and SuperScript II reverse transcriptase (Thermo Fisher) in accordance with the protocol of the manufacturer. Reverse transcription (RT) reactions containing total volumes of 20 µl were conducted by implementing a PrimeScript RT reagent kit (Takara, Japan), and cDNA was maintained at −20 °C until utilization. The DNAzol^™^ Reagent kit (Thermo Fisher) was implemented for the isolation of DNA from cells in accordance with the instructions of the manufacturer. Cells were lysed through agitating the cultivation plate and slowly pipetting the lysate into an assay tube. Following the precipitation of DNA with 100% ethanol, 75% ethanol was employed for DNA rinses. The DNA was air-dried, and in 20 μl of DNase-free water was dissolved, and quantified by applying a NanodropTM spectrophotometer (Thermo Fisher) at 280 and 260 nm. If A260/A280 > 1.8, the DNA was maintained at −20 °C until utilization.

### Quantitative RT-PCR (qRT-PCR)

For quantitative PCR, 2 µl of diluted RT product was blended with 10 µl of 2× SYBR Master mix (Toyobo, Japan), 1 µl each of the reverse and forward primers (10 µM; Supplementary Table 1), and 6 µl of nuclease-free water in a final volume of 20 µl in accordance with the instructions of the manufacturer. Amplification was exerted with the iQ5 quantitative PCR system (Bio-Rad, USA). qRT-PCR was executed in triplicate, comprising nontemplate controls. GAPDH was implemented for normalization of expressions, and 2^−ΔΔCt^ values were normalized to the GAPDH levels. For intranuclear RNA quantification, U6 small nuclear RNA was employed as the internal control.

### Western blot analysis

Western blotting was implemented for analyzing total cellular protein in the specimens. The samples were separated with 10% sodium dodecylsulfate-polyacrylamide gel electrophoresis (SDS-PAGE) and transferred onto PVDF membranes (Millipore, USA). The incubation of membranes was exerted with primary antibodies (Supplementary Table 2) during the night hours at 4 °C. Subsequently, the membranes were rinsed and incubated with secondary antibodies for 1 h (1:2000 for all, Proteintech). At last, the PVDF membranes were exposed to western blotting assessment employing an ECL immunoblotting kit (Beyotime Institute of Biotechnology) according to the protocol of the manufacturer. Each band was normalized regarding its corresponding GAPDH band.

### Cell proliferation/clone assay

Cell proliferation assessments were conducted by implementing a Cell Counting Kit-8 (CCK8) (Dojindo Molecular Technologies, Japan) in accordance with the protocol of the manufacturer. Hepatoma cells were plated in the 96-well plates in triplicate at a density of 5000 cells per well after transfection. Each well contained 120 μL culture medium and optical density were assessed at 450 nm at the demonstrated time points including 0 h, 24 h, 48 h and 72 h. For the assay of colony formation, 1000 cells were seeded in the plates containing 6 wells for 10 days, and colonies were fixed and stained with crystal violet solution. Each cell line was evaluated in three parallel replicates.

### Cell apoptosis analysis

For apoptosis analysis, flow cytometric analysis was selected for quantitative comparison. Briefly, the cells after transfection were incubated with H_2_O_2_ for 12 h before harvested through trypsinization. By taking advantage of Annexin V and propidium iodide (China Sinopharm International), the cultures were double-stained in the dark for 30 min. Cultures were collected and scrutinized for cell apoptosis by employing a flow cytometer (FACScan) equipped with CellQuest 3.3 computer program.

### Transwell assay

Briefly, quantitative cell migration assessments were exerted by employing the plates containing 24 wells comprising chambers with 8 μm polycarbonate filters. Hepatoma cells (5 × 10^5^) were seeded in serum-free milieu and permitted to translocate toward complete milieu supplied with 10% fetal bovine serum. The cells that invaded through the membrane into the lower surface following 24 h were fixed, stained and counted at 37 °C. Microscopically, 200 × representative fields of view were selected for cell quantification.

### Wound healing assay

Wounds were created by employing a 100 μl plastic pipette tip in incubation wells (1 × 10^6^). After a 24 h incubation, wound sizes were accessed and imaged. The cell migration area was measured between dashed regions by (0 h–24 h)/0 h as the healing percentage.

### RNA decay assay

We performed an RNA decay assay by using actinomycin D (ActD) (Sigma-Aldrich) to verify RNA stability. Hepatoma cells were harvested from 6-well plates at 0, 1, and 2 h for qRT-PCR after adding 15 μg ActD. The relative expression levels of each mRNA at 0 h were normalized to 1.

### Immunohistochemistry (IHC)/Immunofluorescence (IF)

Subcutaneous xenograft tissues were sectioned to make 4 μm thick slices. The sections of the tissue were de-paraffinized in xylene and re-hydrated with ethanol, and endogenous peroxidase was inactivated for 10 min by 0.3% hydrogen peroxide. All processes were exerted by employing an UltraSensitiveTM S-P kit (Maixinbio, China) in accordance with the instructions of the manufacturer. An RBM47-specific antibody (Proteintech) (Supplementary Table 2) was implemented at a ratio of 1:100. For immunofluorescence, immobilized slices of hepatoma cells fixed with polyformaldehyde were incubated with a primary antibody against RBM47 (Supplementary Table 2) overnight at 4 °C with lenient agitation. To detect the nucleus, the nuclear fluorescent dye 4’,6-diamidino-2-phenylindole (DAPI) (Sigma, Lyon, France) was used.

### RNA immunoprecipitation (RIP) analysis

After RBM47^Flag^ fusion protein transfection in HCCLM3 cells, the binding between RBM47 and UPF1 mRNA was explored by employing the RNA-Binding Protein Immunoprecipitation Kit (EMD Millipore, USA) according to the instructions of the manufacturer. The cells were lysed, and the resulting solutions of lysis were subsequently incubated with antibody against Flag or isotype control IgG. The complexes of RNA-protein were immunoprecipitated with protein A agarose beads, and the RNA was extracted and purified for subsequent qRT-PCR or sequencing.

### Chromatin immunoprecipitation (ChIP) analysis

The HCCLM3 cells were crosslinked for 10 min with 1% formaldehyde by inverting flasks at ambient temperature and quenched for 5 min with 0.125 M glycine. The cell pellets were rinsed for several times in PBS and then kept at −80 °C. The pellets were lysed for 10 min in the buffer of lysis. Following the centrifugation, the supernatant was left, and the pellet was lysed in the buffer of lysis and exposed to sonication. The incubation of sheared chromatin was fulfilled with the primary antibody bound to PierceTM Protein A/G Agarose Beads (Thermo Fisher) during the night hours, succeeded by elution and reverse cross-linking during the night hours at 65 °C. TE buffer (1 mM ethylenediaminetetraacetic acid (EDTA), 10 mM Tris-HCl) was added to DNA elution buffer succeeded by RNase processing (0.5 mg/mL) for 30 min at 37 °C and proteinase K processing (0.3 mg/mL) for 1 h at 51 °C, and subsequently the DNA was isolated and purified for qRT-PCR or sequencing.

### DNA/RNA sequencing

After chromatin/RNA immunoprecipitation, DNA/RNA library construction was performed by DNA/cDNA repair, including loading Base A and sequencing connector. Qubit 2.0 (Invitrogen) was used for quality inspection before sequencing on an Illumina sequencer. We compared the raw data to obtain comprehensive transcript information, quantify gene expression, and analyze Gene Ontology (GO) and Kyoto Encyclopedia of Genes and Genomes (KEGG) pathways. Meanwhile, genome-wide de novo peak was carried out to test the binding preference of proteins on DNA/RNA. Reads ≥ 10 was defined as valid sequence and RPKM was calculated for sequence screening. Homer de novo Motif analysis was performed to predict preference binding sites. Enriched motifs with top 30 significance would be listed and sorted by *P* values to match to target sequences.

### Luciferase reporter assays

To verify the activity of the 3’UTR and promoter, a luciferase reporter was used for indirect quantitative analysis. Briefly, luciferase activity was evaluated by applying a Dual Luciferase Assay Kit (Promega, USA) in accordance with the instructions of the manufacturer. The wild-type 3’UTR and promoter of UPF1 (containing putative binding sites plus 60 nucleotides upstream and downstream) were embedded into a pGL3-basic vector (Promega, USA) downstream or upstream of the luciferase reporter gene, creating the wild-type or mutant plasmid. All fluorescence intensities were assessed implementing a luminometer (Promega, USA).

### Pulldown assay

The oligonucleotides containing biotin on the 5’ nucleotide of the sense strand were used in the pulldown assays. The UPF1 promoter or its 3’UTR probe (wild type and mutation) was incubated with HCCLM3 cell lysates and streptavidin magnetic beads for DNA or RNA pulldown. The beads were rinsed, and the proteins were eluted and resolved by PAGE. The fragments of the gel were excised and scrutinized through western blotting.

### Animal experiments

Male athymic 4-week-old BALB/c nude mice were acquired from the Animal Center of the Chinese Academy of Medical Sciences (Beijing, China) and were kept in a specific pathogen-free facility. HCCLM3 cells stably transfected with RBM47/empty plasmid with fluorescence labeling were harvested from the plates containing 6 wells and suspended at 2–4 × 10^6^cells/mL. Tumor growth assays were performed by subcutaneously injecting the cells into the armpits of the mice. Tumor growth curves and tumor weights were calculated for quantitative comparison. For the observation of metastases, 1 × 10^6^cells/mL suspensions were injected into the tail veins of 10 nude mice (5 in each group, 4 weeks old). At the end of the 5th week, the lung fluorescence intensities in vivo were tested by an IVIS® Lumina III System (PerkinElmer, USA) to quantitatively compare metastasis.

### Statistical analysis

To compare the continuous variables between groups, student’s *t*-test was executed. Statistical discrepancies between groups were scrutinized employing SPSS 22.0 computer program (IBM, Chicago, IL, USA), and plots were generated using GraphPad Prism 6.0 (GraphPad Software, USA).

## Supplementary information

Supplementary Table 1

Supplementary Table 2

Supplementary Table 3

Supplementary Table 4

Supplementary Figure 1

Supplementary Figure 2

Supplementary Figure 3

Original Data File

## Data Availability

Data supporting the findings of this study are available within the paper and its Supplementary Data files.
