# Supplementary material for: RBM47 inhibits hepatocellular carcinoma progression by targeting UPF1 as a DNA/RNA regulator
Source: Cell Death Discov. 2022 Jul 14;8:320. doi: 10.1038/s41420-022-01112-3 (PMC9279423; doi:10.1038/s41420-022-01112-3)
Supplement: Supplementary file 1 — Supplementary Table 1 [file 41420_2022_1112_MOESM1_ESM.docx]

**Supplementary Table S1.** Primer sequences used in this research.

| **Gene** | **Sequence (5'-3')** |
| --- | --- |
| RBM47-F | ATCAGCAATCCTTGGCTCAC |
| RBM47-R | CCTTGGGATTCCTCTGTTCA |
| UPF1-F | AAGGTATGGCGTCATCATTGTGG |
| UPF1-R | CCGTGGCTTGCTGAACTGC |
| UPF1 (for ChIP)-F | ATGGTCTCGATCTCCTG |
| UPF1 (for ChIP)-R | AAAGCTGCTTGGAACTC |
| GAPDH-F | GGTATCGTGGAAGGACTCAT |
| GAPDH-R | CCTTGCCCACAGCCTTG |
| Pre-UPF1 (23I24E)-F | CCCCATCCTGTCTGCTCCG |
| Pre-UPF1 (23I24E)-R | CTGATGGACTAAGCCACGTT |
| Pre-UPF1 (24E)-F | GTCACTGGCCCTGATCCGAA |
| Pre-UPF1 (24E)-R | ACCGAAAGCCCTAGAGCAA |
| U6-F | GCTCTCGCATCGCAGCA |
| U6-R | CTCGGCTAGCGCTACTC |
