# Supplementary material for: RBM47 inhibits hepatocellular carcinoma progression by targeting UPF1 as a DNA/RNA regulator
Source: Cell Death Discov. 2022 Jul 14;8:320. doi: 10.1038/s41420-022-01112-3 (PMC9279423; doi:10.1038/s41420-022-01112-3)
Supplement: Supplementary file 2 — Supplementary Table 2 [file 41420_2022_1112_MOESM2_ESM.docx]

**Supplementary Table S2.** Antibodies used in this study.

| **Antibody** | **Company** | **Cat No.** | **Western Blot** | **IHC** | **IF** |
| --- | --- | --- | --- | --- | --- |
| GAPDH | Proteintech | 60004-1-Ig | 1:2000 |  |  |
| RBM47 | Proteintech | 23902-1-AP | 1:2000 |  |  |
| RBM47 | Thermo Fisher | PA5-52282 |  | 1:100 | 1:200 |
| Ki-67 | Santa Cruz | sc-15402 |  | 1:200 |  |
| Flag Tag | BUSABIO | CSB-MA000021M0m | 1:2000 |  |  |
| Smad7 | Proteintech | 25840-1-AP | 1:1000 |  |  |
| Bax | Proteintech | 50599-2-Ig | 1:5000 |  |  |
| Bcl-2 | Proteintech | 12789-1-AP | 1:2000 |  |  |
| E-cadherin | Proteintech | 20874-1-AP | 1:2000 |  |  |
| N-cadherin | Proteintech | 22018-1-AP | 1:2000 |  |  |
| Vimentin | Proteintech | 10366-1-AP | 1:2000 |  |  |
