# Supplementary material for: RBM47 inhibits hepatocellular carcinoma progression by targeting UPF1 as a DNA/RNA regulator
Source: Cell Death Discov. 2022 Jul 14;8:320. doi: 10.1038/s41420-022-01112-3 (PMC9279423; doi:10.1038/s41420-022-01112-3)
Supplement: Supplementary file 3 — Supplementary Table 3 [file 41420_2022_1112_MOESM3_ESM.docx]

**Supplementary Table S3.** Enriched motifs with top 30 significance according to Homer de novo Motif analysis based on RIP-seq.

| Rank | Motif | *P* | % of Targets | % of Targets |
| --- | --- | --- | --- | --- |
| 1 | 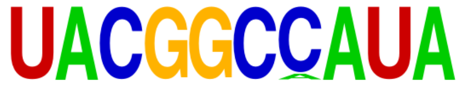 | 1e-21 | 1.32% | 0.00% |
| 2 | 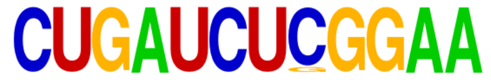 | 1e-21 | 1.32% | 0.00% |
| 3 | 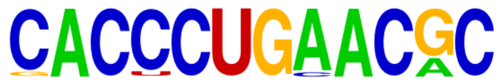 | 1e-21 | 1.47% | 0.01% |
| 4 | 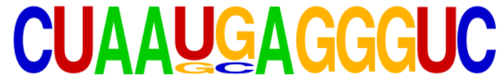 | 1e-20 | 1.47% | 0.01% |
| 5 | 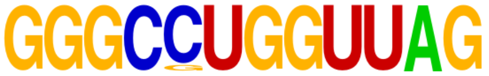 | 1e-19 | 1.17% | 0.00% |
| 6 | 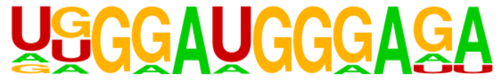 | 1e-13 | 1.62% | 0.04% |
| 7 | 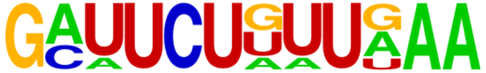 | 1e-13 | 1.03% | 0.01% |
| 8 | 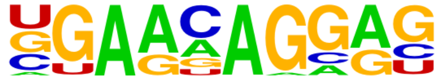 | 1e-12 | 19.24% | 10.10% |
| 9 | 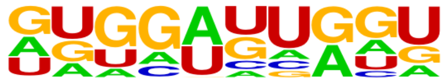 | 1e-11 | 4.41% | 0.87% |
| 10 | 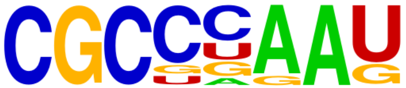 | 1e-11 | 3.23% | 0.44% |
| 11 | 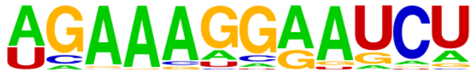 | 1e-11 | 6.61% | 2.01% |
| 12 | 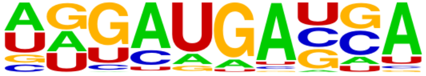 | 1e-11 | 21.88% | 12.54% |
| 13 | 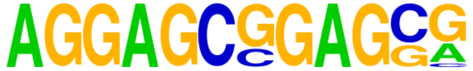 | 1e-10 | 1.17% | 0.03% |
| 14 | 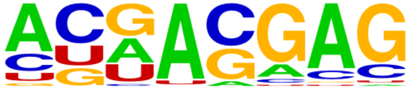 | 1e-10 | 13.07% | 6.24% |
| 15 | 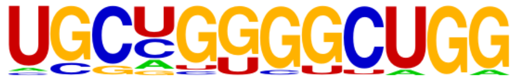 | 1e-9 | 2.64% | 0.37% |
| 16 | 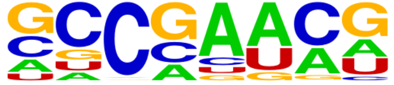 | 1e-9 | 7.34% | 2.70% |
| 17 | 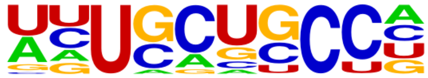 | 1e-9 | 13.80% | 7.08% |
| 18 | 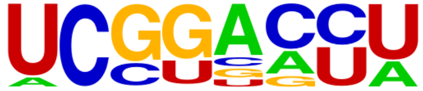 | 1e-8 | 6.02% | 2.01% |
| 19 | 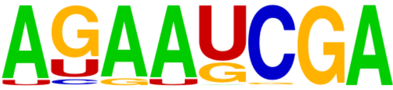 | 1e-8 | 4.41% | 1.16% |
| 20 | 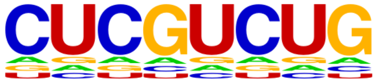 | 1e-8 | 1.32% | 0.07% |
| 21 | 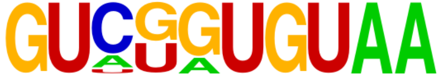 | 1e-8 | 0.73% | 0.01% |
| 22 | 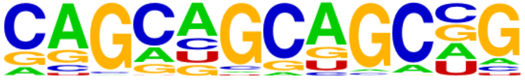 | 1e-8 | 0.59% | 0.00% |
| 23 | 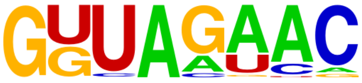 | 1e-7 | 2.79% | 0.57% |
| 24 | 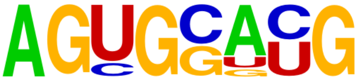 | 1e-6 | 3.82% | 1.13% |
| 25 | 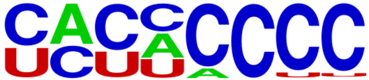 | 1e-6 | 13.66% | 7.82% |
| 26 | 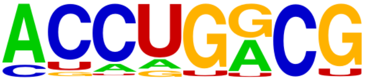 | 1e-6 | 6.61% | 2.83% |
| 27 | 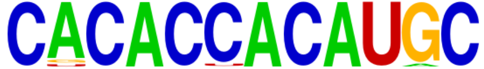 | 1e-6 | 0.44% | 0.00% |
| 28 | 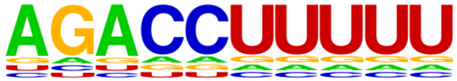 | 1e-5 | 0.59% | 0.01% |
| 29 | 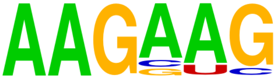 | 1e-5 | 43.32% | 34.86% |
| 30 | 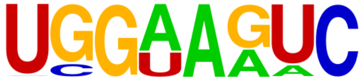 | 1e-4 | 6.31% | 3.20% |
