# Supplementary material for: RBM47 inhibits hepatocellular carcinoma progression by targeting UPF1 as a DNA/RNA regulator
Source: Cell Death Discov. 2022 Jul 14;8:320. doi: 10.1038/s41420-022-01112-3 (PMC9279423; doi:10.1038/s41420-022-01112-3)
Supplement: Supplementary file 4 — Supplementary Table 4 [file 41420_2022_1112_MOESM4_ESM.docx]

**Supplementary Table S4.** Enriched motifs with top 30 significance according to Homer de novo Motif analysis based on ChIP-seq.

| Rank | Motif | *P* | % of Targets | % of Targets |
| --- | --- | --- | --- | --- |
| 1 | 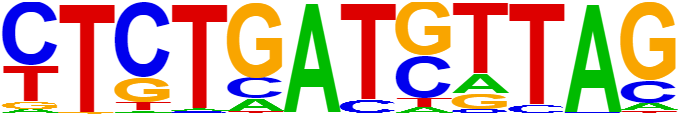 | 1e-1788 | 4.63% | 0.33% |
| 2 | 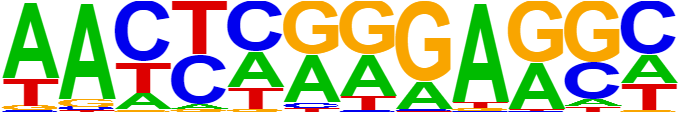 | 1e-1788 | 30.78% | 14.97% |
| 3 | 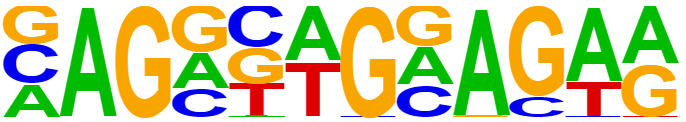 | 1e-1564 | 35.71% | 19.65% |
| 4 | 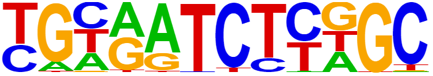 | 1e-1461 | 37.23% | 21.37% |
| 5 | 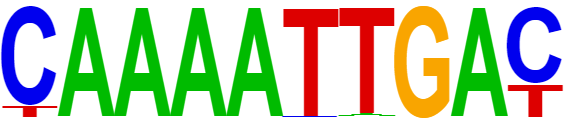 | 1e-1329 | 4.53% | 0.51% |
| 6 | 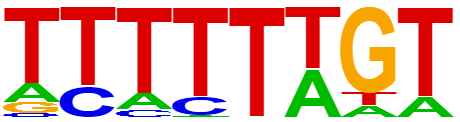 | 1e-1262 | 45.40% | 29.48% |
| 7 | 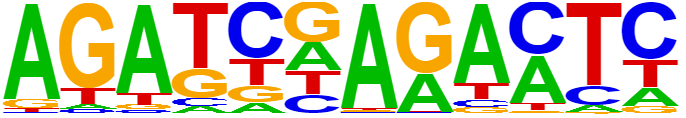 | 1e-1155 | 21.09% | 10.17% |
| 8 | 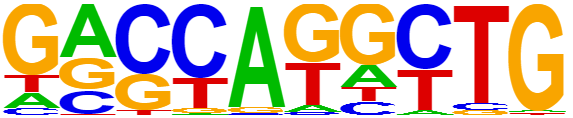 | 1e-1031 | 35.11% | 21.83% |
| 9 | 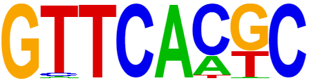 | 1e-998 | 21.89% | 11.38% |
| 10 | 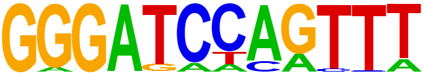 | 1e-988 | 4.30% | 0.68% |
| 11 | 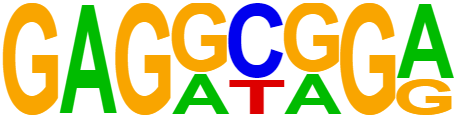 | 1e-975 | 40.68% | 27.01% |
| 12 | 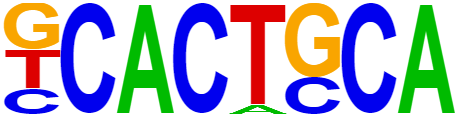 | 1e-955 | 31.31% | 19.06% |
| 13 | 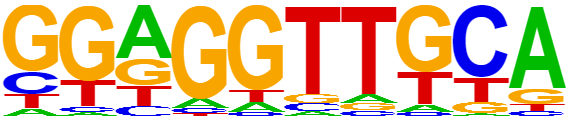 | 1e-942 | 28.02% | 16.43% |
| 14 | 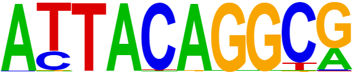 | 1e-820 | 28.36% | 17.39% |
| 15 | 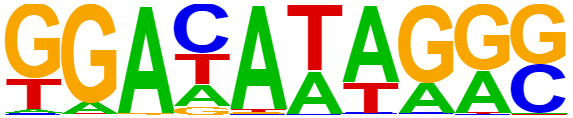 | 1e-773 | 4.77% | 1.08% |
| 16 | 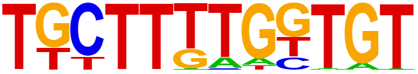 | 1e-771 | 2.76% | 0.33% |
| 17 | 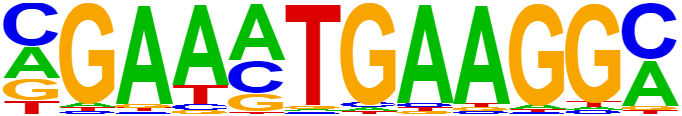 | 1e-716 | 1.93% | 0.15% |
| 18 | 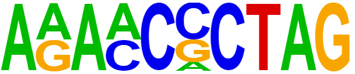 | 1e-682 | 2.86% | 0.43% |
| 19 | 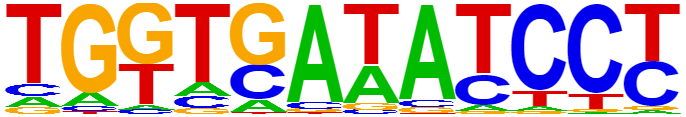 | 1e-674 | 16.52% | 8.81% |
| 20 | 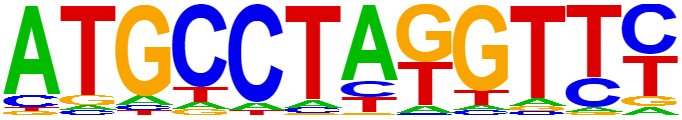 | 1e-654 | 1.67% | 0.11% |
| 21 | 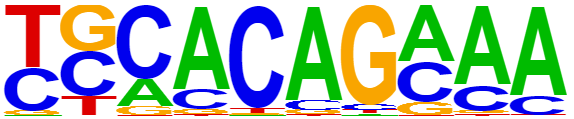 | 1e-618 | 3.30% | 0.65% |
| 22 | 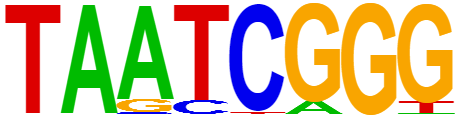 | 1e-602 | 36.78% | 26.21% |
| 23 | 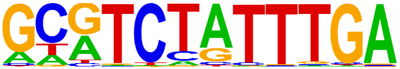 | 1e-576 | 1.36% | 0.08% |
| 24 | 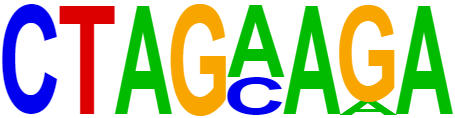 | 1e-566 | 4.42% | 1.23% |
| 25 | 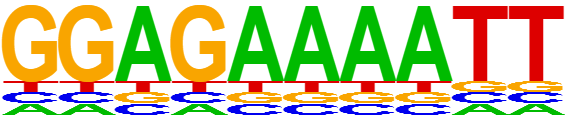 | 1e-460 | 1.71% | 0.22% |
| 26 | 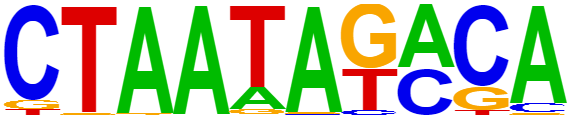 | 1e-447 | 1.88% | 0.28% |
| 27 | 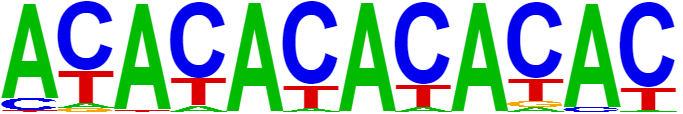 | 1e-447 | 47.74% | 37.92% |
| 28 | 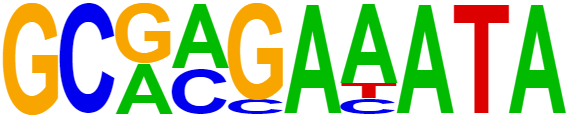 | 1e-443 | 1.34% | 0.13% |
| 29 | 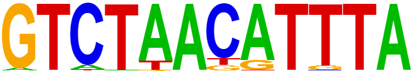 | 1e-392 | 1.23% | 0.12% |
| 30 | 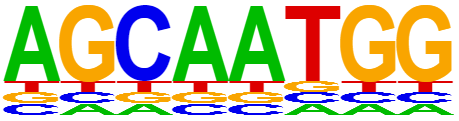 | 1e-339 | 2.74% | 0.78% |
