# Supplementary material for: RBM47 inhibits hepatocellular carcinoma progression by targeting UPF1 as a DNA/RNA regulator
Source: Cell Death Discov. 2022 Jul 14;8:320. doi: 10.1038/s41420-022-01112-3 (PMC9279423; doi:10.1038/s41420-022-01112-3)

**Figure S1. RBM47 expression in hepatoma cells**.

1. RBM47 mRNA was detected in different hepatoma cell lines by qRT-PCR. Error bars are SD (n = 3). After knockdown in Huh7 cells and overexpression in HCCLM3 cells by transient transfection, RBM47 expression was tested by (B) qRT-PCR and (C) Western blot. Error bars are SD (n = 3). ****P* < 0.001.


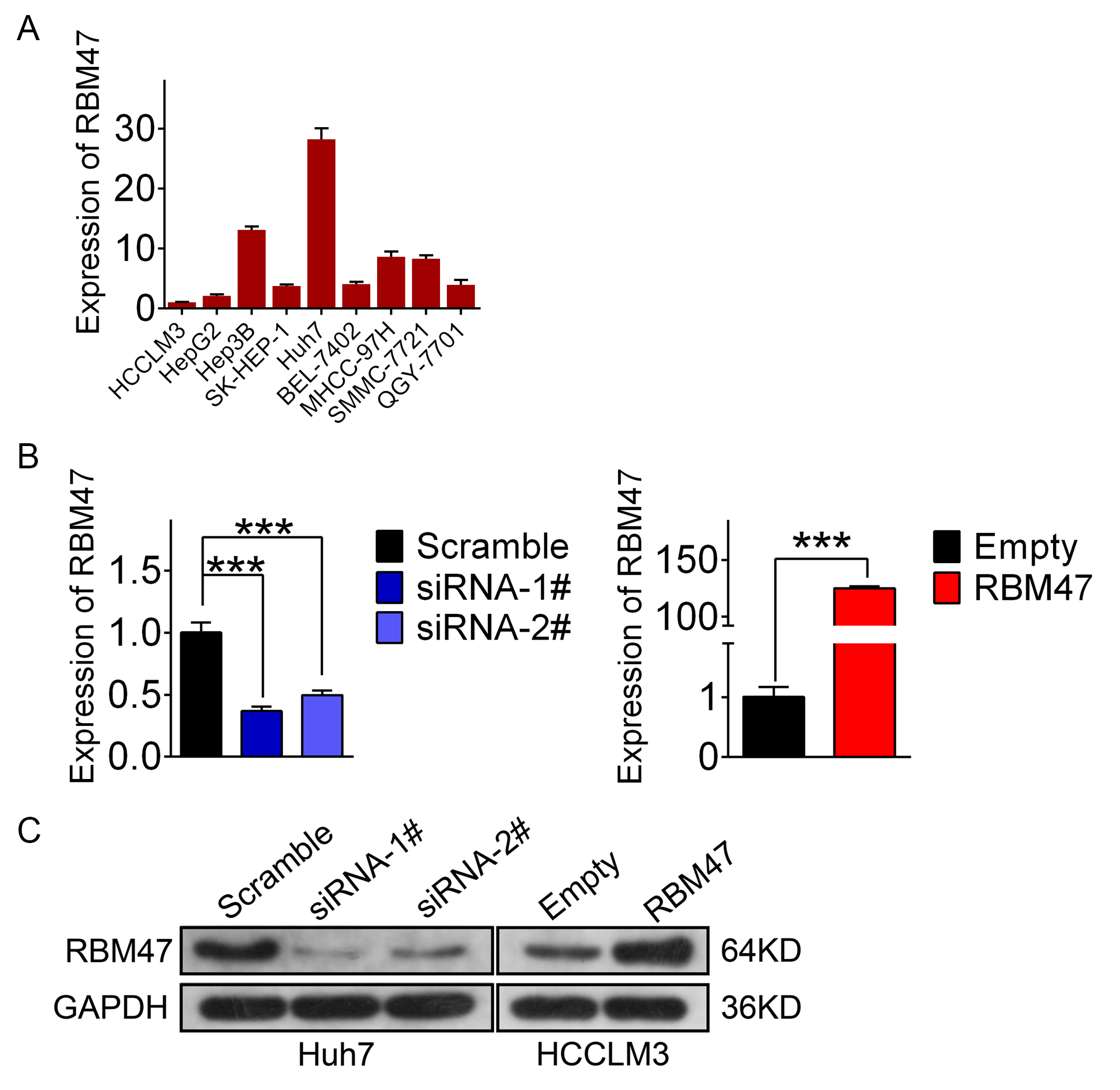

Supplement: Supplementary file 5 — Supplementary Figure 1 [file 41420_2022_1112_MOESM5_ESM.docx]
