# Supplementary material for: RBM47 inhibits hepatocellular carcinoma progression by targeting UPF1 as a DNA/RNA regulator
Source: Cell Death Discov. 2022 Jul 14;8:320. doi: 10.1038/s41420-022-01112-3 (PMC9279423; doi:10.1038/s41420-022-01112-3)

**Figure S2. Stable transfection with RBM47 in HCCLM3 cells.**

After stable transfection with the RBM47 vector in vitro, RBM47 expression was tested by (A) qRT-PCR and (B) Western blot. Error bars are SD (n = 3). ****P* < 0.001.


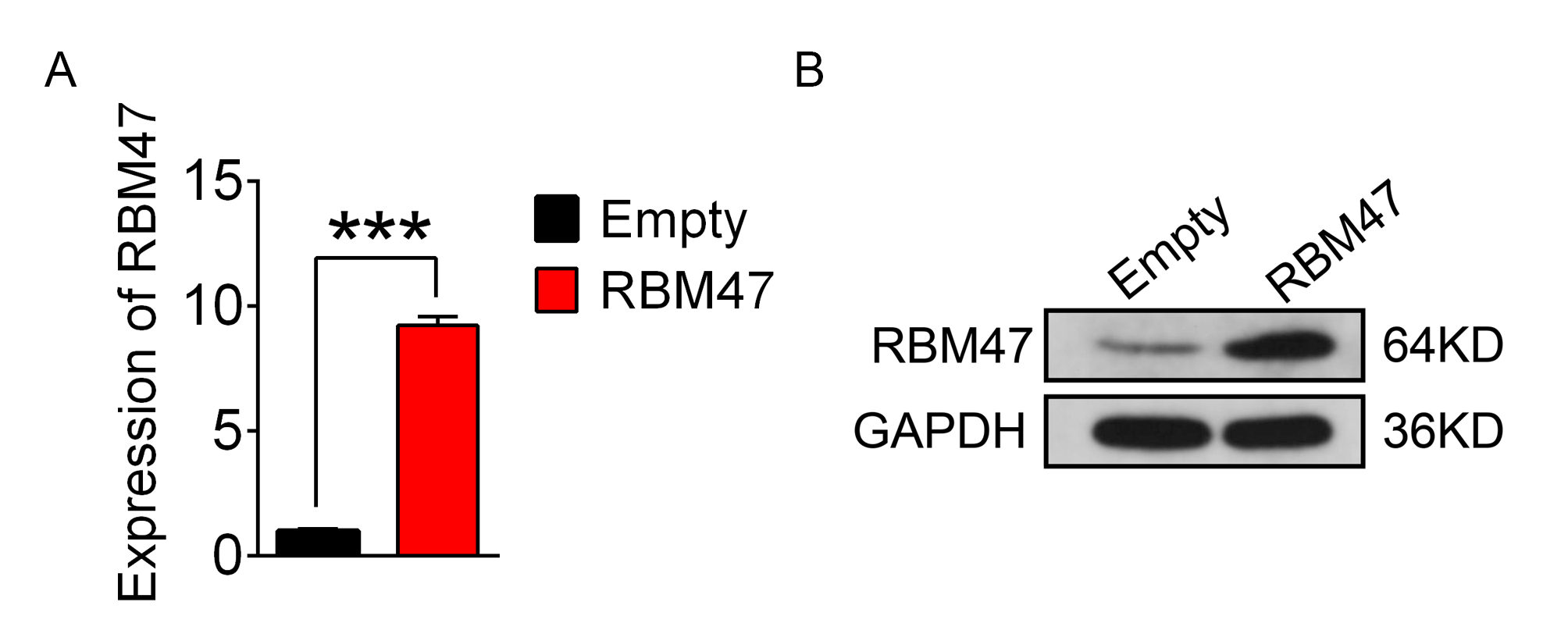

Supplement: Supplementary file 6 — Supplementary Figure 2 [file 41420_2022_1112_MOESM6_ESM.docx]
