# Supplementary material for: RBM47 inhibits hepatocellular carcinoma progression by targeting UPF1 as a DNA/RNA regulator
Source: Cell Death Discov. 2022 Jul 14;8:320. doi: 10.1038/s41420-022-01112-3 (PMC9279423; doi:10.1038/s41420-022-01112-3)

**Figure S3. The expression of UPF1 in hepatoma cells.**

With transient transfection with UPF1 siRNA or vector, (A) qRT-PCR and (B) Western blot detected the expression of UPF1 in Huh7 cells with UPF1 silencing and in HCCLM3 cells with UPF1 expression. Error bars are SD (n = 3). ****P* < 0.001.


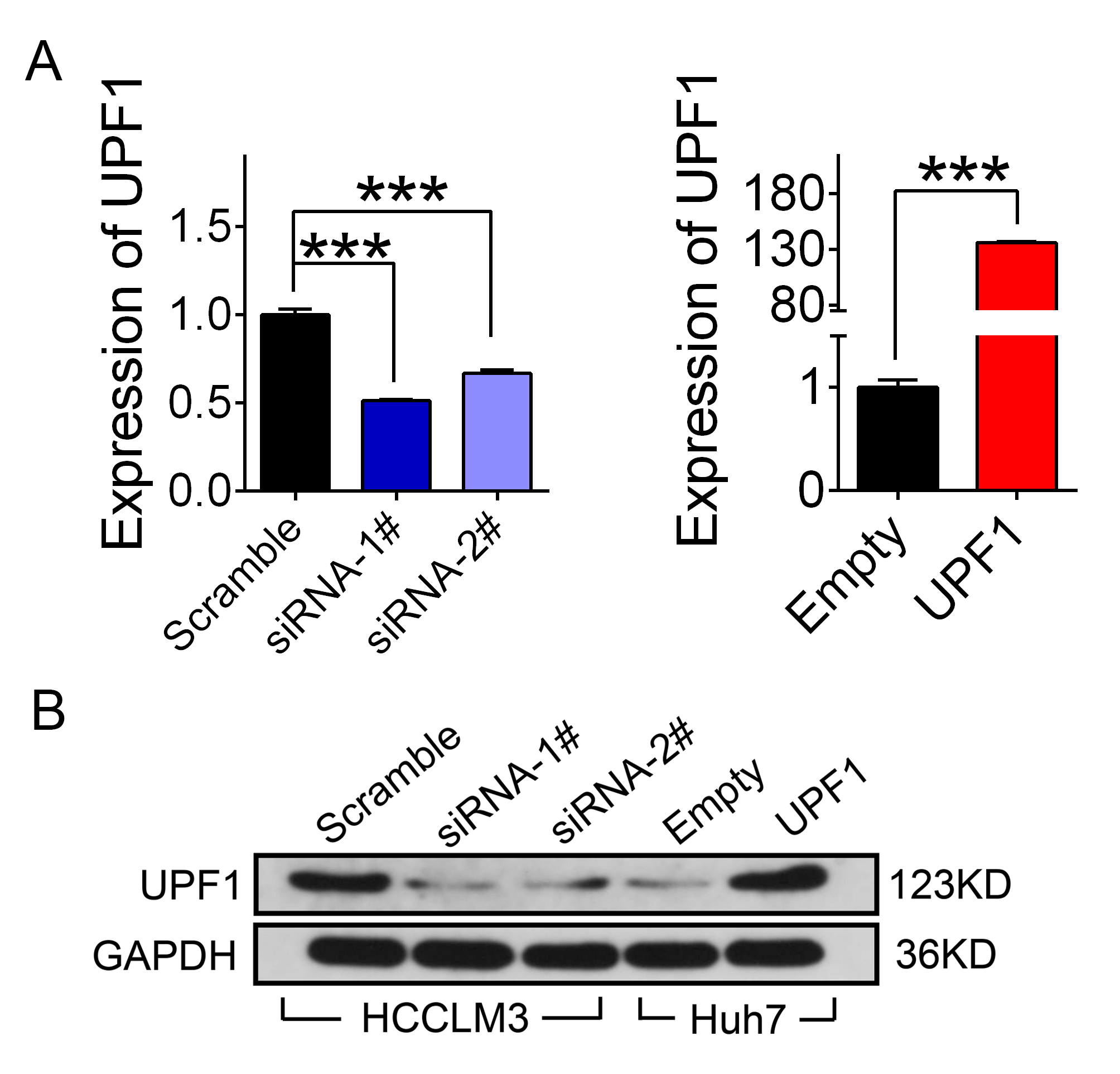

Supplement: Supplementary file 7 — Supplementary Figure 3 [file 41420_2022_1112_MOESM7_ESM.docx]
