# Supplementary material for: RBM47 inhibits hepatocellular carcinoma progression by targeting UPF1 as a DNA/RNA regulator
Source: Cell Death Discov. 2022 Jul 14;8:320. doi: 10.1038/s41420-022-01112-3 (PMC9279423; doi:10.1038/s41420-022-01112-3)

Figure 4G

(UPF1)

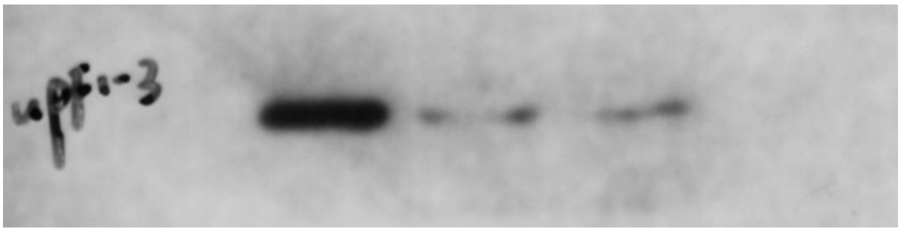

(GAPDH)

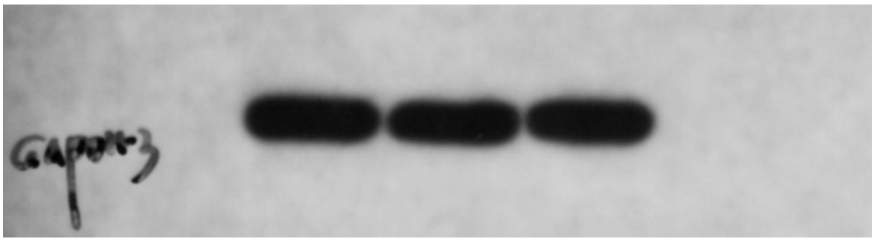

(UPF1)

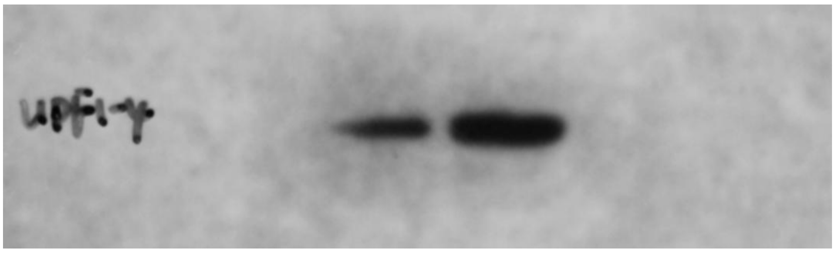

(GAPDH)

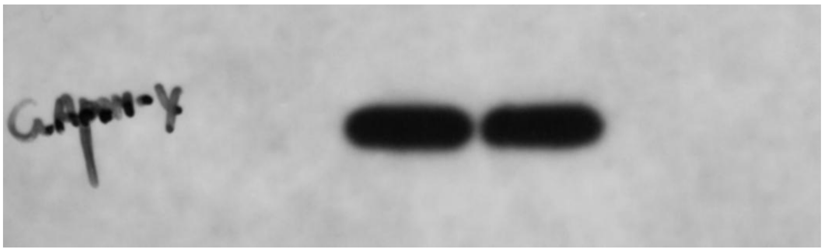

Figure 5D

(Smad7)

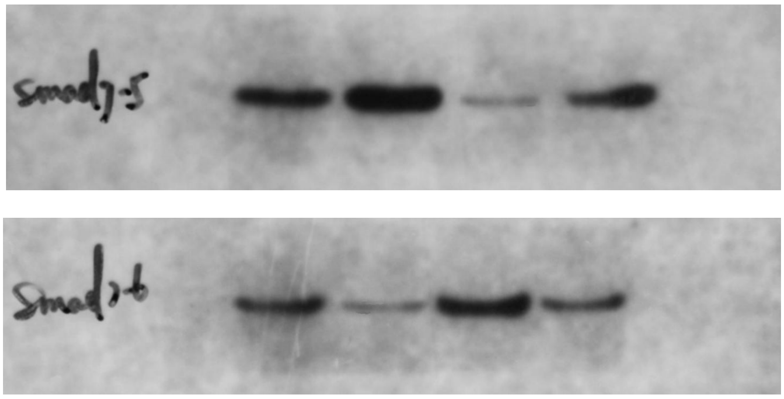

(Bax)

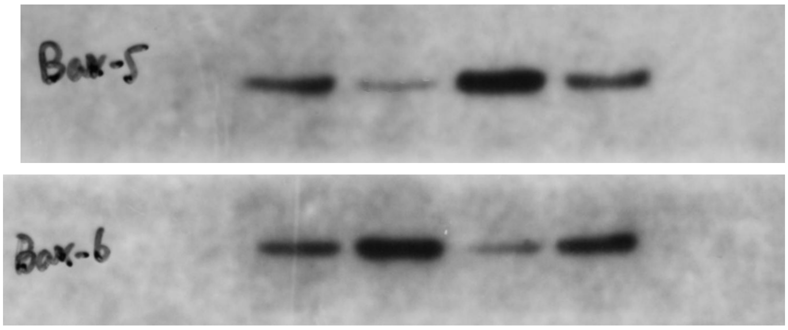

(Bcl-2)

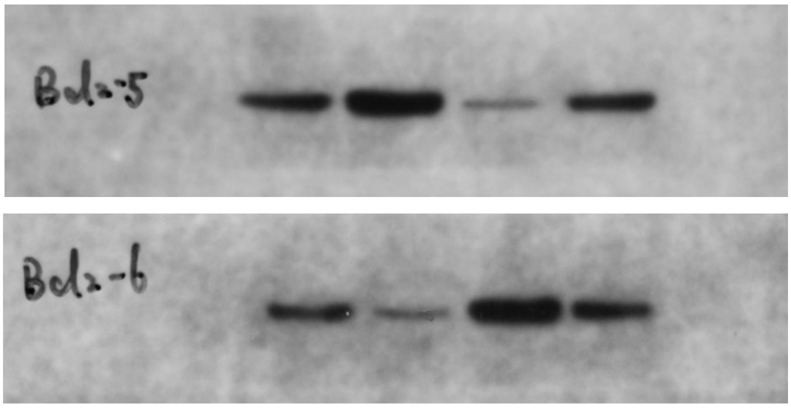

(E-cadherin)

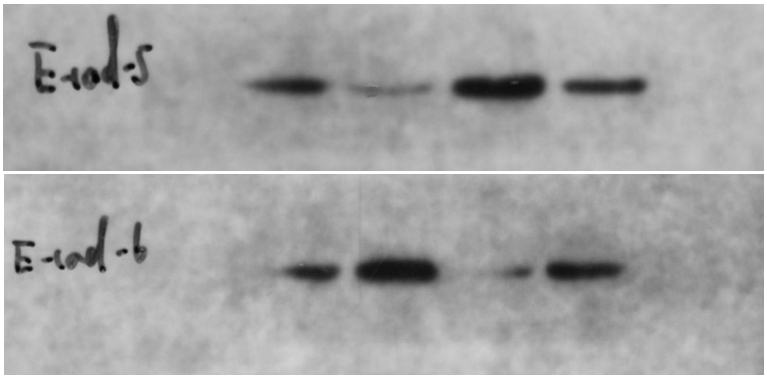

(N-cadherin)

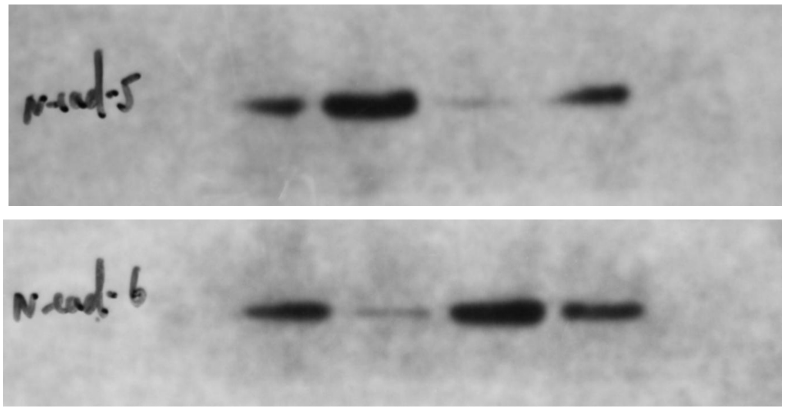

(Vimentin)

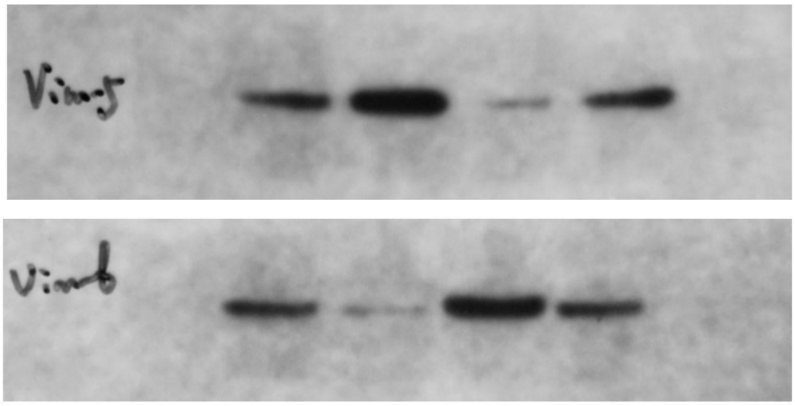

(GAPDH)

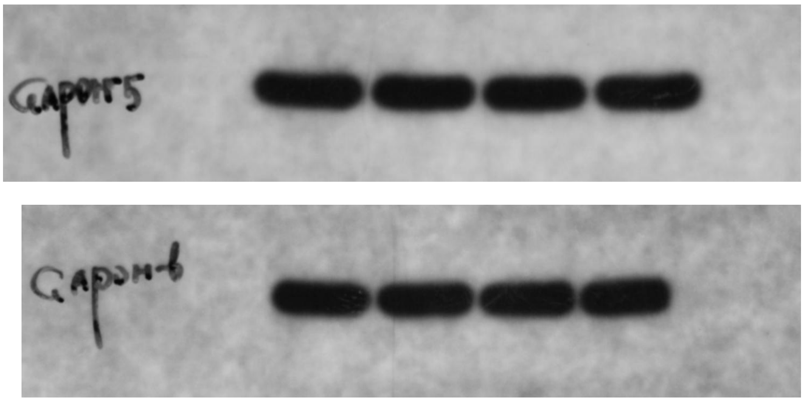

Figure 6F

(RBM47-FLAG)

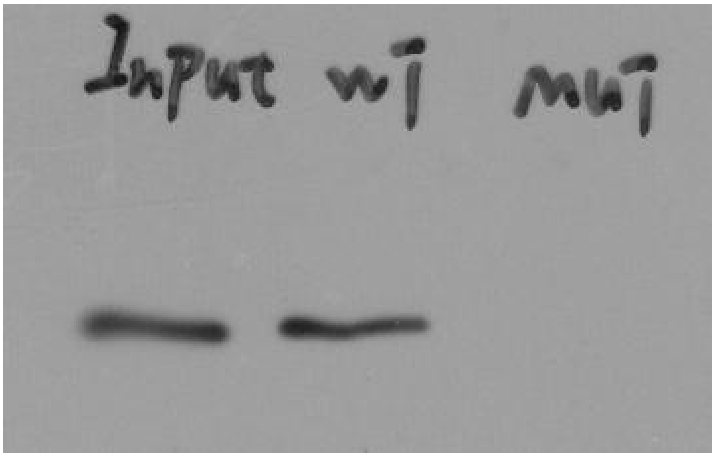

Figure 8F

(RBM47-FLAG2)

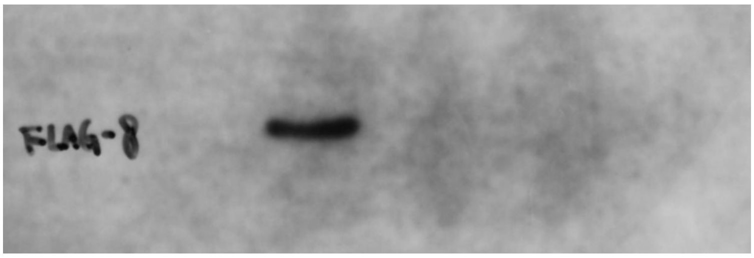

(RBM47-FLAG1)

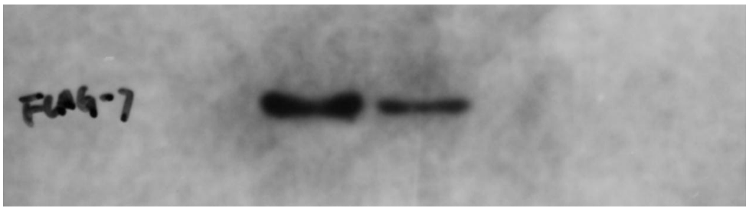

Figure S1C

(RBM47)

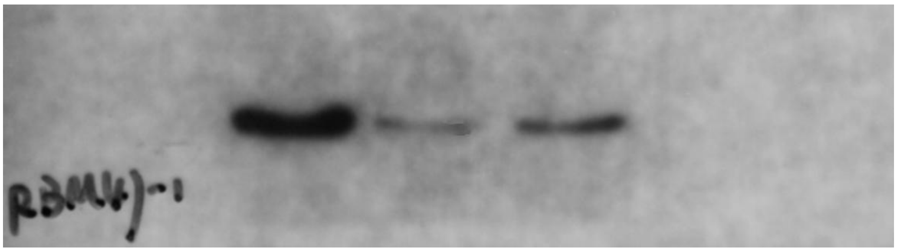

(GAPDH)

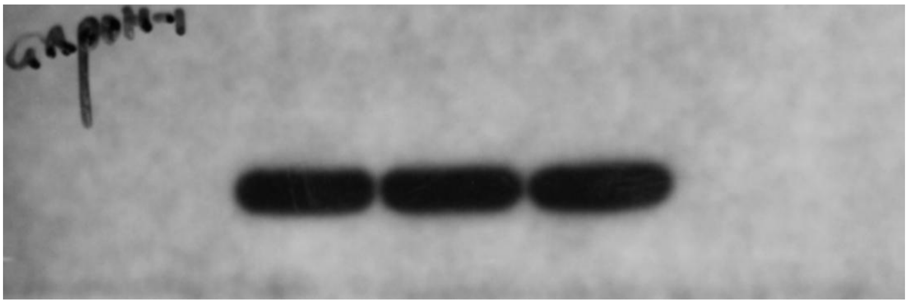

(RBM47)

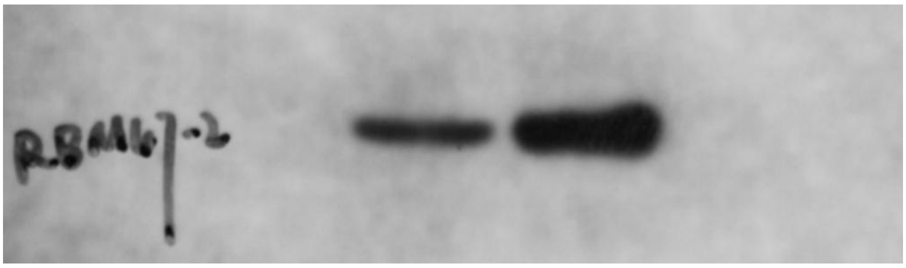

(GAPDH)

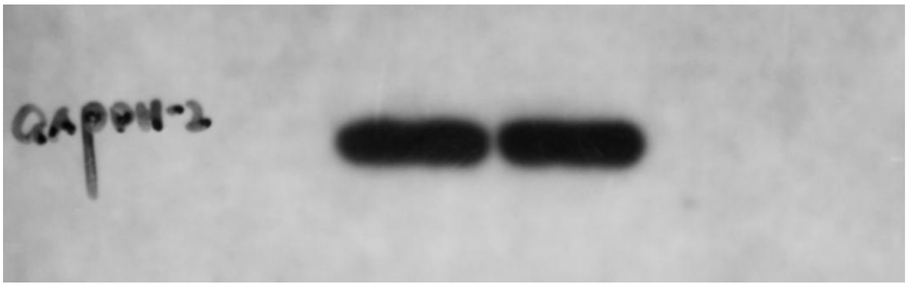

Figure S2B

(RBM47)

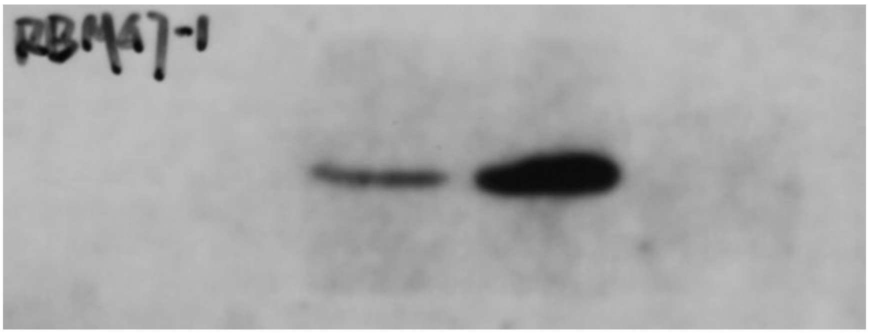

(GAPDH)

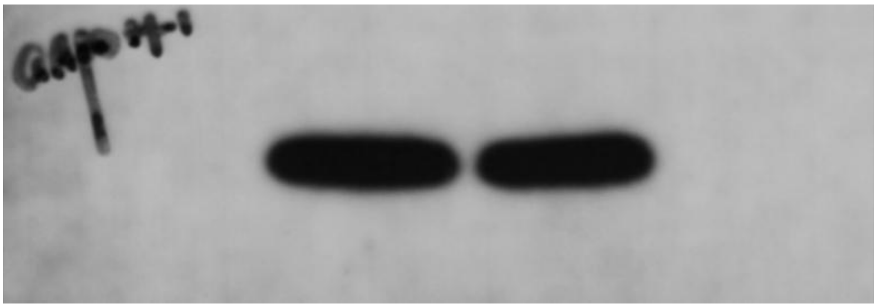

Figure S3B

(UPF1)

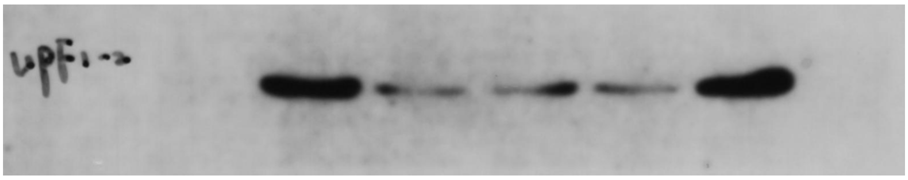

(GAPDH)

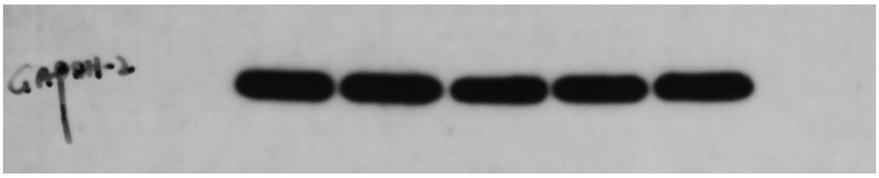

Supplement: Supplementary file 8 — Original Data File [file 41420_2022_1112_MOESM8_ESM.pdf]
